# Ribosome profiles and riboproteomes of healthy and Potato virus A‐ and *Agrobacterium*‐infected *Nicotiana benthamiana* plants

**DOI:** 10.1111/mpp.12764

**Published:** 2018-12-06

**Authors:** Katri Eskelin, Markku Varjosalo, Janne Ravantti, Kristiina Mäkinen

**Affiliations:** ^1^ Department of Microbiology, Faculty of Agriculture and Forestry University of Helsinki PO Box 56 FI‐00014 Finland; ^2^ Molecular and Integrative Biosciences Research Programme, Faculty of Biological and Environmental Sciences University of Helsinki PO Box 56 FI‐00014 Finland; ^3^ Institute of Biotechnology University of Helsinki PO Box 65 FI‐00014 Finland

**Keywords:** *Agrobacterium tumefaciens*, asymmetrical flow field‐flow fractionation, bioinformatics, LC‐MS/MS, Potato virus A, riboproteome, ribosomes, *Nicotiana benthamiana*

## Abstract

*Nicotiana benthamiana* is an important model plant for plant–microbe interaction studies. Here, we compared ribosome profiles and riboproteomes of healthy and infected *N. benthamiana* plants. We affinity purified ribosomes from transgenic leaves expressing a FLAG‐tagged ribosomal large subunit protein RPL18B of *Arabidopsis thaliana*. Purifications were prepared from healthy plants and plants that had been infiltrated with *Agrobacterium tumefaciens* carrying infectious cDNA of Potato virus A (PVA) or firefly luciferase gene, referred to here as PVA‐ or *Agrobacterium*‐infected plants, respectively. Plants encode a number of paralogous ribosomal proteins (r‐proteins). The *N. benthamiana* riboproteome revealed approximately 6600 r‐protein hits representing 424 distinct r‐proteins that were members of 71 of the expected 81 r‐protein families. Data are available via ProteomeXchange with identifier PXD011602. The data indicated that *N. benthamiana* ribosomes are heterogeneous in their r‐protein composition. In PVA‐infected plants, the number of identified r‐protein paralogues was lower than in *Agrobacterium*‐infected or healthy plants. *A. tumefaciens* proteins did not associate with ribosomes, whereas ribosomes from PVA‐infected plants co‐purified with viral cylindrical inclusion protein and helper component proteinase, reinforcing their possible role in protein synthesis during virus infection. In addition, viral NIa protease‐VPg, RNA polymerase NIb and coat protein were occasionally detected. Infection did not affect the proportions of ribosomal subunits or the monosome to polysome ratio, suggesting that no overall alteration in translational activity took place on infection with these pathogens. The riboproteomic data of healthy and pathogen‐infected *N. benthamiana* will be useful for studies on the specific use of r‐protein paralogues to control translation in infected plants.

## Introduction

The ribosomal 40S subunit is composed of 18S ribosomal RNA (rRNA) and 33 ribosomal proteins (r‐proteins). This number also includes ribosome‐associated receptor for activating C kinase 1 (RACK1) (Pisarev *et al*., 2008; Sengupta *et al*., 2004). The 60S subunit has 5S, 5.8S and 25‐26S rRNAs, and 48 r‐proteins. The translation of r‐proteins occurs in the cytoplasm and, subsequently, they are transported into the nucleolus for subunit assembly. Final maturation takes place after export to the cytoplasm, where the acidic P‐proteins P0, P1, P2 and P3 bind to the 60S subunit to form a motile stalk (Szick *et al*., 1998). The two subunits combine during translation initiation to form a translation‐competent 80S ribosome. Polysomes consist of varying numbers of 80S ribosomes, which all synthesize the same protein. Global down‐regulation of translation initiation, which causes alterations in the abundance of 40S and 60S subunits, monosomes and polysomes, may occur in various stress conditions (Bailey‐Serres *et al*., 2009).

In plants, multiple paralogous r‐protein genes that share high sequence similarity encode each r‐protein (Barakat *et al*., 2001; Hummel *et al*., 2015; Whittle and Krochko, 2009). The majority of them are transcribed (Barakat *et al*., 2001; Whittle and Krochko, 2009), translated (Schippers and Mueller‐Roeber, 2010) and incorporated into ribosomes (Carroll *et al*., 2008; Chang *et al*., 2005; Giavalisco *et al*., 2005; Hummel *et al*., 2012, 2015). The *A. thaliana* genome contains 242 functional r‐protein genes (Barakat *et al*., 2001; Chang *et al*., 2005; Hummel *et al*., 2015). Their incorporation into ribosomes would allow ~10^34^ compositionally different ribosome assemblies (Hummel *et al*., 2012). Transcribed r‐protein genes vary with the tissue type and developmental stage (McIntosh and Bonham‐Smith, 2006; Whittle and Krochko, 2009), as well as with external stimuli (Wang *et al*., 2013). Importantly, mutation and knockdown studies indicate that certain paralogues may have specialized functions in plant growth and development (Degenhardt and Bonham‐Smith, 2008; Horiguchi *et al*., 2012; Schippers and Mueller‐Roeber, 2010). The r‐proteins of *A. thaliana* range from 3.4 to 44.7 kDa in size (Barakat *et al*., 2001; Chang *et al*., 2005).

The r‐proteins are mostly located on the surface of ribosomes (Klinge *et al*., 2012). It has been suggested that they serve as a docking station for ribosome‐associated proteins that assist in translation regulation (Xue and Barna, 2012). Accordingly, minor changes in the ribosome surfaces caused by heterogeneity in the r‐protein sequences could affect the repertoire of ribosome‐associated proteins bound on the ribosome surfaces. Proteomic studies of ribosomes have identified non‐ribosomal proteins that co‐purify with cytosolic plant ribosomes (Carroll *et al*., 2008; Chang *et al*., 2005; Hummel *et al*., 2012). For example, the well‐known RACK1 binds to the 40S subunit in a 1 : 1 ratio and interacts with proteins involved in translational regulation (Adams *et al*., 2011; Kundu *et al*., 2013; Nilsson *et al*., 2004).

In this work, we studied the protein composition of ribosomes of *N. benthamiana*. *N. benthamiana* is widely used as a model plant to study plant–microbe interactions (Goodin *et al*., 2008), as it is readily infected with numerous plant pathogens, including viruses, bacteria and fungi. We also investigated the effects of two plant pathogens, *A. tumefaciens* and Potato virus A (PVA, family *Potyviridae*), on *N. benthamiana* riboproteome and translational activity. Potyviruses form a large group of positive‐stranded RNA viruses (reviewed in Ivanov *et al*., 2014). Potyvirus infections induce the transcription of r‐protein genes (Alfenas‐Zerbini *et al*., 2009; Dardick, 2007; Yang *et al*., 2007), suggesting that specialized ribosomes or certain r‐proteins might be required during infection. *A. tumefaciens* is a Gram‐negative soil bacterium belonging to the family *Rhizobiaceae* (reviewed in Tarkowski and Vereecke, 2014). In contrast with potyviruses, *A. tumefaciens *infection represses r‐protein expression (Ditt *et al*., 2006). The proteomic investigation and annotation of healthy, *A. tumefaciens*‐ and PVA‐infected *N. benthamiana* ribosomes, which are reported here, will be an important source of information for further studies of translational control in pathogen‐infected plants.

## Results

**Figure 1:**
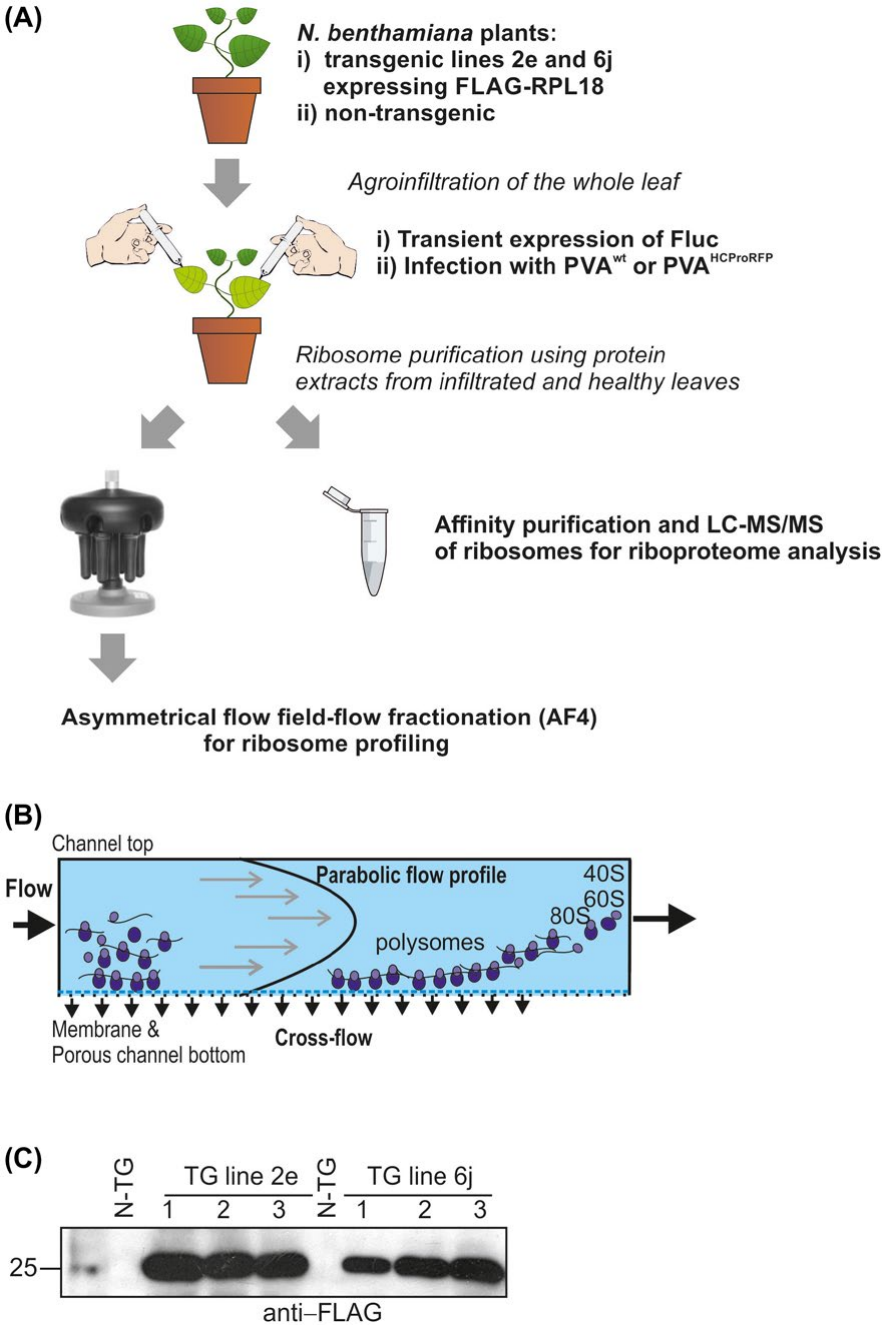
Workflow used in this study. (A) Ribosome purification procedures. Non‐transgenic or transgenic *Nicotiana benthamiana* plants expressing FLAG‐tagged RPL18 from *Arabidopsis thaliana* were infected with Potato virus A (PVA) through agroinfiltration (PVA‐infected plants). In addition, plants were agroinfiltrated with firefly luciferase (Fluc) expression construct (*Agrobacterium*‐infected plants) or were left non‐treated (healthy plants). Infiltrated leaves were collected at 3 and 4 days post‐infection (dpi) and employed to purify the ribosomes using ultracentrifugation or anti‐FLAG immunoaffinity resin. Further separation of ribosomal subunits, monosomes and polysomes for ribosome profiling was achieved by asymmetrical flow field‐flow fractionation (AF4). Affinity‐purified ribosomes were further analysed by liquid chromatography‐tandem mass spectrometry (LC‐MS/MS). (B) The operating principle of AF4. Sample components are separated gently without stationary phase based on their hydrodynamic sizes by the application of two simultaneous flows: channel flow and cross‐flow. In a default elution mode, small sample components elute before the larger ones. (C) Western blot analysis with anti‐FLAG antibodies showing the expression of FLAG‐tagged RPL18 in transgenic *N. benthamiana* lines 2e and 6j. FLAG‐tagged RPL18 levels were comparable in both transgenic lines. [Colour figure can be viewed at wileyonlinelibrary.com]

The workflow used to obtain samples for ribosome profiles and riboproteome studies is presented in Fig. 1A. Extracts were pelleted by ultracentrifugation at 170 000 ***g*** (P170K samples) from healthy, *Agrobacterium‐* and PVA‐infected *N. benthamiana* plants and further fractionated by asymmetrical flow field‐flow fractionation (AF4) to obtain the ribosome profiles. The operating principle of AF4 is presented in Fig. 1B. Affinity purification of ribosomes to obtain riboproteomes was performed via a FLAG‐tag, which has been proven to be successful in studies of the *Arabidopsis* translatome and proteome (Hummel *et al*., 2012, 2015; Mustroph *et al*., 2009; Zanetti *et al*., 2005). We used FLAG‐resin to immunopurify *N. benthamiana* ribosomes from transgenic lines 2e and 6j which both express FLAG‐tagged *Arabidopsis* RPL18B equally (Fig. 1C)*.* Professor Moffett (Université de Sherbrooke, QC, Canada) kindly provided these plants. *Arabidopsis* FLAG‐RPL18 incorporates into *N. benthamiana* ribosomes (Pitkänen *et al*., 2014).

**Figure 2:**
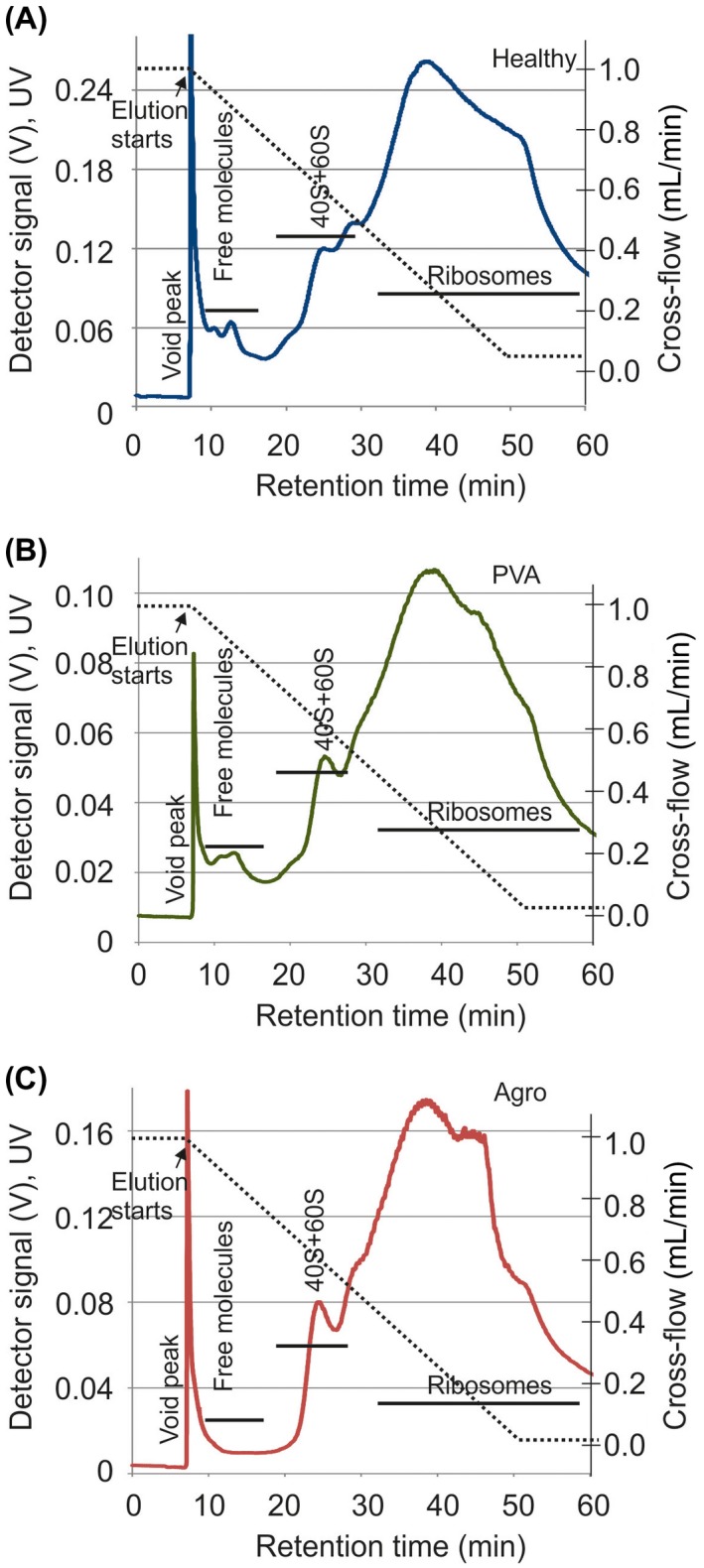
Asymmetrical flow field‐flow fractionation (AF4) reveals that the ribosome profiles are alike in healthy and infected plants. P170K ribosome profiles were analysed from healthy (A), Potato virus A (PVA)‐infected (B) and *Agrobacterium*‐infected (C) *N. benthamiana* plants. Representative fractograms are shown. Samples were focused for 6 min prior to transition to a linearly decaying cross‐flow gradient from 1 mL/min to 0.05 mL/min (broken line). The peak at ~6 min is the void peak. Retention times for ribosomes were obtained from Pitkänen et al. (2014). The elution of sample components was followed using a UV detector by monitoring the intensity (V) at 254 nm (full lines). [Colour figure can be viewed at wileyonlinelibrary.com]

### Ribosome profiles of healthy and pathogen‐infected plants are alike

Translational inhibition caused by stress is often evidenced by a decrease in polysomes, as well as an increase in 80S monosomes, 40S and 60S subunit levels (Bailey‐Serres, 1999; Fennoy *et al*., 1998; Groppo and Palmenberg, 2007; Hummel *et al*., 2015). Followed by the separation of the 40S and 60S subunits, 80S monosomes and polysomes by A4F, the UV fractograms obtained were similar (Fig. 2), indicating that the studied pathogens did not induce changes in the subunit, monosome and polysome ratio.

Stress may induce the formation of 80S ribosomes devoid of mRNA (Martin and Hartwell, 1970). To test whether infection induced the formation of 80S ribosomes free of mRNA, we performed KCl treatment for P170K samples prior to AF4. Such ribosomes can be dissociated into subunits using high KCl concentrations (Martin and Hartwell, 1970), because they are not stabilized by mRNA, tRNA and nascent polypeptide. The observed changes in the UV fractograms were modest and comparable for ribosomes of healthy and infected plants (Fig. S1, see Supporting Information). Heat shock causes a significant reduction in the overall translational activity *in planta* (Matsuura *et al*., 2010). In contrast with ribosomes of pathogen‐infected plants, ribosomes that were extracted from plants exposed to heat shock showed a clear increase in ribosomal subunits that were sensitive to KCl (Fig. S1C). We conclude that the studied pathogens do not induce the formation of 80S ribosomes lacking mRNAs and do not alter the overall translational activity.

### PVA helper component proteinase (HCPro), cytoplasmic inclusion protein (CI), nuclear inclusion protein a (NIa) and coat protein (CP) are present in the ribosome‐enriched fractions

**Figure 3:**
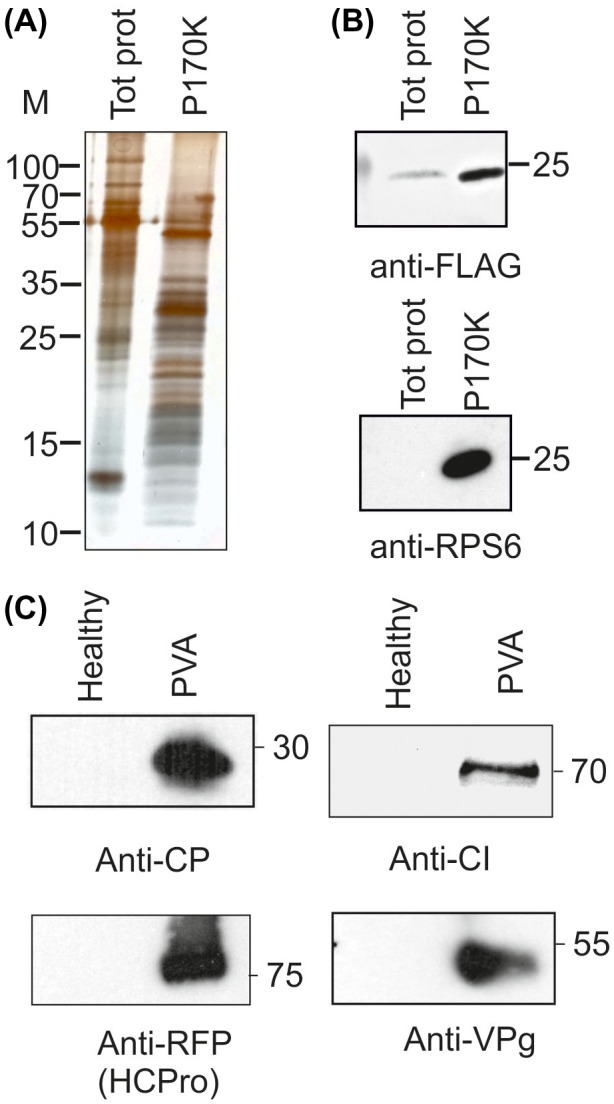
The Potato virus A (PVA) proteins helper component proteinase (HCPro), cytoplasmic inclusion protein (CI), nuclear inclusion protein a (NIa) and coat protein (CP) are present in the ribosome‐enriched P170K samples. (A) Protein pattern of P170K samples in silver‐stained sodium dodecylsulfate‐polyacrylamide gel electrophoresis (SDS‐PAGE) showed enrichment of proteins below 55 kDa when compared with the total protein sample. (B) The presence of ribosomes in P170K samples was verified by western blotting with antisera recognizing RPL18 from the 60S subunit (anti‐FLAG) and RPS6 from the 40S subunit. (C) PVA proteins CI, NIa and CP were detected with their corresponding antisera, and HCPro with anti‐RFP antiserum recognizing RFP‐tagged HCPro. [Colour figure can be viewed at wileyonlinelibrary.com]

Several PVA proteins may be ribosome associated. We studied the presence of viral proteins in ribosomal P170K pellets derived from *N. benthamiana* lines 2e and 6j infected with PVA^HCPro‐RFP^ at 4 days post‐infection (dpi). Enrichment of proteins below 55 kDa could be observed in the P170K sample (Fig. 3A). As the calculated sizes of the r‐proteins vary in general between 3 and 55 kDa, this suggests the enrichment of ribosomes. The presence of r‐proteins from both subunits was verified with western blots that detected FLAG‐RPL18 (60S) and RPS6 (40S) (Fig. 3B). We used PVA‐specific antisera to show the presence of several PVA proteins in P170K samples (Fig. 3C). PVA CP and CI were readily detected. Antisera against viral genome‐linked protein (VPg) detected the full‐length NIaPro‐VPg fusion of approximately 55 kDa, whereas monomeric VPg was below the detection limit (data not shown). The presence of PVA HCPro was demonstrated as an HCPro‐RFP fusion using red fluorescent protein (RFP) antibodies.

As PVA CP, VPg, HCPro and CI occur in virus particles or cytoplasmic inclusions (Lohmus *et al*., 2016; Roberts *et al*., 1998), it is possible that these macromolecular structures co‐sedimented with the ribosome‐associated fractions. To ensure that the outcomes represented association with ribosomes, we carried out electron microscopy imaging of P170K pellets to study whether any high‐molecular‐weight aggregates were present in the samples (Fig. S2, see Supporting Information). We only observed spherical structures that were similar to ribosomes detected in a commercial wheatgerm extract *in vitro* translation mixture. This suggested that P170K pellets were devoid of viral particles as well as CI‐ and HCPro‐formed inclusions, and that these proteins were detected in the P170K samples because of their ribosome association.

**Figure 4:**
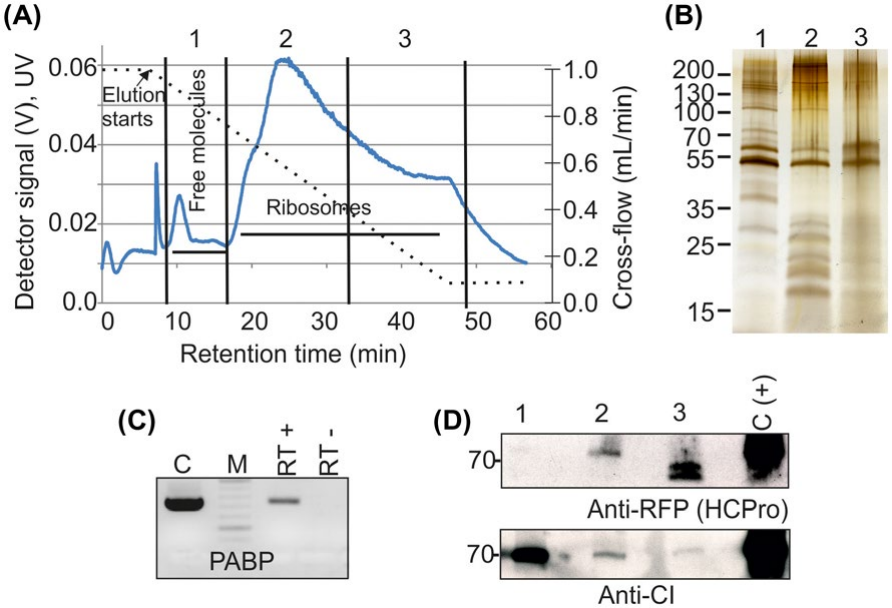
The Potato virus A (PVA) cytoplasmic inclusion protein (CI) and helper component proteinase (HCPro) associate with polysomes. (A) AF4 fractionation of P170K samples derived from PVA‐infected *Nicotiana benthamiana* plants. The P170K sample was focused for 6 min prior to elution with a linearly decaying cross‐flow from 1 mL/min to 0.05 mL/min (broken line). The peak at approximately 6 min is the void peak. Elution of molecules was followed by monitoring of the UV detector intensity (V) at 254 nm (full line). Fractions 1–3 for further analyses contained the eluates collected during the indicated retention times. (B) Soluble proteins and small protein complexes enriched in fraction 1, 40S and 60S subunits, 80S monosomes and small polysomes enriched in fraction 2 and large polysomes enriched in fraction 3 were concentrated and analysed by sodium dodecylsulfate‐polyacrylamide gel electrophoresis (SDS‐PAGE) and silver staining. (C) Reverse transcription‐polymerase chain reaction (RT‐PCR) verified that pooled fractions 2 and 3 contained mRNA (RT+). C, positive PCR control in which total RNA was used as a template for cDNA synthesis; RT–, first‐strand synthesis reaction without reverse transcriptase. PABP, poly(A)‐binding protein. (D) Concentrated AF4 samples 1–3 were probed for the presence of PVA HCPro or CI using anti‐red fluorescent protein (anti‐RFP) or anti‐CI antibodies and enhanced chemiluminescence (ECL) detection. Total plant cell lysate from PVA‐infected plants expressing RFP‐tagged HCPro served as a positive control [C(+)]. [Colour figure can be viewed at wileyonlinelibrary.com]

We analysed the ribosomal P170K samples from PVA^HCPro‐RFP^‐infected plants further with AF4 (Fig. 4A), and collected fractions that contained components that were smaller than ribosomal subunits (fraction 1), as well as ribosomal subunits, 80S monosomes and polysomes (fractions 2–3), according to the retention times analysed in Pitkänen *et al*. (2014). Sodium dodecylsulfate‐polyacrylamide gel electrophoresis (SDS‐PAGE) demonstrated the enrichment of proteins with the expected size range of r‐proteins in fractions 2 and 3 (Fig. 4B). Reverse transcription‐polymerase chain reaction (RT‐PCR) analysis verified the presence of various mRNA molecules in these fractions, indicating that they contained polysomes that were active in translation. Of these, the presence of poly(A)‐binding protein (PABP) mRNA was demonstrated (Fig. 4C). We also probed the fractions with antibodies against CI and RFP to detect RFP‐HCPro (Fig. 4D). The signal for HCPro was most intense in fraction 3, indicating that it is abundantly associated with actively translating ribosomes. However, the size of the detected RFP‐HCPro was smaller than expected, indicating that some proteolytic cleavage may have occurred. Full‐sized HCPro‐RFP fusion was detected in fraction 2 that contained small polysomes, monosomes and ribosomal subunits. PVA CI was detected in all AF4 fractions. However, the majority was in fraction 1 which contained sample components that were smaller than ribosomal subunits.

### Neither *Agrobacterium* nor PVA infection modifies translation factors in *N. benthamiana*

The 5′‐untranslated region of potyviral RNA is covalently linked to VPg and functions as an internal ribosome entry site (IRES; as reviewed in Ivanov *et al*., 2014; Revers and Garcia, 2015). Picornaviruses, sharing these properties with potyviruses, inhibit cellular cap‐dependent translation by proteolytic cleavage of eIF4E/iso4E, eIF4G/eIFiso4G or PABP (Bushell and Sarnow, 2002; Fitzgerald and Semler, 2009). Here, samples were collected at 0, 1 and 2 dpi to determine whether similar *N. benthamiana* factors were targeted by PVA proteases on infection. We did not observe any changes in the accumulation or size of eIF4E/iso4E (Fig. S3, see Supporting Information). Western blot detection of eIF4G/eIFiso4G with anti‐eIF4G antibody did not succeed because of several non‐specific recognitions (data not shown). We observed some reduction in PABP accumulation at 1 and 2 dpi, but detected no proteolytic cleavage products (Fig. S3). PABP transcription has been shown to decrease in the early stages of PVA infection in potatoes (*Solanum tuberosum* ssp. *andigena*) (Vuorinen *et al*., 2010).

### *N. benthamiana* genome contains 611 r‐protein genes

To examine the total number of r‐protein genes present in healthy and infected *N. benthamiana* ribosomes, a genome‐wide bioinformatics analysis was carried out. There were several search approaches, as outlined in Experimental procedures, and the combined outcomes identified 611 r‐proteins. In addition to the 563 previously annotated r‐proteins that included RACK1 and P‐protein candidates, re‐analysis of the *N. benthamiana* sequence data combined with the liquid chromatography‐mass spectrometry (LC‐MS/MS) data (see below) allowed us to identify 38 additional r‐protein candidates (Table S2, see Supporting Information). These 611 sequences included 229 genes encoding the 33 putative 40S subunit proteins and 382 genes encoding the 48 putative 60S subunit proteins. This indicated that *N. benthamiana* contained an average of eight paralogues (611/81) for each r‐protein. The highest number of paralogues, 17, was observed for RPS8 (Table S2). The annotated *N. benthamiana* sequence has no annotations for RPL41 (Bombarely *et al*., 2012). We found 74 sequences sharing some similarity with *Arabidopsis rpl*41, but homology between them was generally low (~28%) and they were longer than expected (Table S2). The published *N. benthamiana* genome had only single annotations for *rps*29 and *rpl*40 (Bombarely *et al*., 2012), whereas plant genomes usually contain more than two paralogous r‐protein genes (Barakat *et al*., 2001; Whittle and Krochko, 2009). We re‐analysed the *N. benthamiana* transcriptome and genome for additional *rps*29 and *rpl*40 sequences, but found none.

We compared similarities among *N. benthamiana, A. thaliana *and *N. sylvestris* r‐proteins (Table S2; Fig. S4, see Supporting Information). *Arabidopsis* r‐proteins of the same family are usually similar in size and share 65%–100% amino acid sequence identity, the majority being close to 100% identical (Barakat *et al*., 2001; Chang *et al*., 2005) (see also Table S2). Many candidate r‐protein genes of *N. benthamiana* showed large amino acid sequence differences, including truncations, insertions, extensions or internal deletions, which were not present in the corresponding *Arabidopsis* proteins (Tables S2 and S3, see Supporting Information). For the sake of reliability, only r‐protein sequences sharing minimally 30% similarity were included in multiple sequence alignments and the generation of the phylogenetic trees (Table S2). The majority of analysed sequences grouped to the corresponding r‐protein families, and r‐proteins of *N. benthamiana* were more similar to those of *N. sylvestris* than *A. thaliana *(Fig. S4A–C)*.* Most analysed *N. benthamiana* r‐protein families had some members with 100% identity, indicating that paralogue‐specific identifications would not always be possible (Table S2). In general, the bioinformatics analyses indicated that the quality of the used r‐protein sequence data allows the identification of only a rudimentary *N. benthamiana* riboproteome, which needs to be further amended when *N*. *benthamiana* genome sequence data will allow.

### Affinity purification yields high‐quality *N. benthamiana* ribosomes for riboproteome analysis

**Figure 5:**
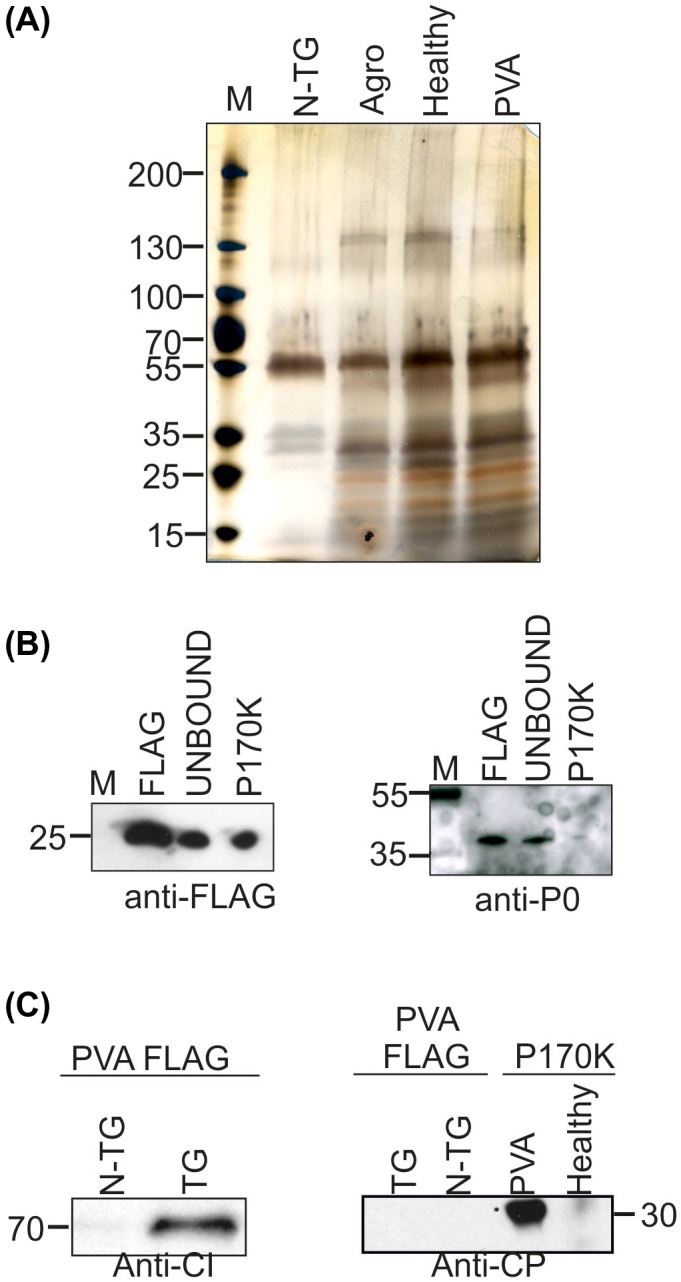
Analysis of the affinity‐purified samples reveals the presence of ribosomes in FLAG‐RPL18 and cytoplasmic inclusion protein (CI) in Potato virus A (PVA)‐infected FLAG‐RPL18 samples. (A) The protein pattern in the silver‐stained sodium dodecylsulfate‐polyacrylamide gel electrophoresis (SDS‐PAGE) gel in lanes containing samples purified from healthy, *Agrobacterium‐* and PVA‐infected transgenic FLAG‐RPL18 plants shows enrichment of proteins of <55 kDa. (B) Western blotting verified the presence of FLAG‐RPL18 and acidic ribosomal protein P0 in the affinity‐purified FLAG‐RPL18 samples. ‘UNBOUND’ refers to those proteins that did not bind to FLAG‐resin during the incubation time. (C) Affinity‐purified ribosomes from PVA‐infected FLAG‐RPL18 or non‐transgenic control plants were probed with antibodies against CI and coat protein (CP). Antibody–antigen complexes were detected with enhanced chemiluminescence (ECL) detection. N‐TG, non‐transgenic; TG, transgenic FLAG‐RPL18; P170K, pellet after ultracentrifugation in sucrose cushion at 170 000 ***g***. [Colour figure can be viewed at wileyonlinelibrary.com]

As we aimed to produce riboproteomic data from high‐quality translationally active ribosomes, FLAG immunopurification was performed according to the workflow (Fig. 1A). Transgenic FLAG‐RPL18 lines 2e and 6j were agroinfiltrated to deliver PVA or firefly luciferase (Fluc)‐encoding plasmids. Controls included untreated healthy FLAG‐RPL18 and PVA‐infected non‐transgenic plants. Agroinfiltrated and healthy leaves looked alike at 3–4 dpi when the samples were collected. Anti‐FLAG antibodies were used for immunopurification, followed by SDS‐PAGE and silver staining. Gels revealed a greater abundance of proteins of <55 kDa in FLAG‐RPL18 than in non‐transgenic extracts (Fig. 5B), which indicates the enrichment of r‐proteins. Western blotting with the anti‐FLAG and anti‐P0 antibodies verified that the bait protein and acidic r‐protein P0 were present in the samples from FLAG‐RPL18 plants (Fig. 5B,C), further verifying the presence of ribosomes. In addition, ribosomes were visible in electron microscopy (EM) analysis (Fig. S2B), and agarose electrophoresis revealed the presence of the expected rRNA species in a comparable ratio with the leaf total RNA (Fig. S5A, see Supporting information); these observations verified the successful purification of ribosomes. We performed RT‐PCR to study the association of varicose, protein kinase CK2, transcription factor bZIP and acidic r‐protein P0 mRNAs with the purified ribosomes (Fig. S5B) to verify the translational activity of the purified ribosomes. Western blotting analysis of P170K samples indicated that several PVA proteins are ribosome associated (see Fig. 3C). We repeated the western blotting analysis with the affinity‐purified ribosomes and verified the association of CI with ribosomes (Fig. 5C). CP was detected only in the P170K sample, but not in the FLAG‐purified sample, questioning its tight association with ribosomes. We concluded that our purified samples contained intact ribosomes that were active in translation and some viral proteins specifically associated with them in PVA‐infected samples. Therefore, these samples were well suited for the analysis of the riboproteomes of healthy and infected *N. benthamiana*.

### Total numbers of identified proteins in healthy and infected *N. benthamiana* riboproteomes are comparable

We were interested in the composition of *N. benthamiana* ribosomes and whether infection induced specific adjustments in this composition. Samples from healthy, *Agrobacterium*‐ and PVA‐infected FLAG‐RPL18 plants and from non‐transgenic PVA‐infected controls were derived from four independent purifications. Purifications 1 and 2 consisted of two biological replicates of the experiment and was performed at 3 dpi. Purifications 3 and 4 were similar to 1 and 2, but were performed at 4 dpi. All samples were analysed in duplicate (technical replicates) using LC‐MS/MS. Thus, we analysed the riboproteomes from 24 FLAG‐RPL18 samples and eight non‐transgenic controls. To avoid losses of proteins during gel electrophoresis or precipitation, eluates from the affinity purifications were subjected to reverse phase chromatography, trypsination and LC‐MS/MS directly. Consequently, FLAG peptide used to elute the ribosomes from the affinity matrix was the most abundant peptide in all samples [peptide matching score (PSM) values of 282–931]. Otherwise, the PSM values of the identified proteins varied in the range 2–12, with the average being ~7. Altogether 10 527 proteins remained after we had filtered out proteins having a PSM value of 1: purification 1, 1726; purification 2, 2800; purification 3, 3336; purification 4, 2775. The total number of protein hits in differently treated samples were comparable: healthy, 3058; *Agrobacterium*‐infected, 3319; PVA‐infected, 3199.

### Identification of non‐specific binders from non‐transgenic controls

LC‐MS/MS analysis of the controls from non‐transgenic plants was carried out to identify proteins that had non‐specific affinity towards the FLAG‐resin. This analysis showed that the majority of the identified ~11 000 protein hits were specific, as the number of non‐specific binders was only 951 (PSM value above 1). They presented 366 distinct protein annotations (Table S4, see Supporting Information). These controls also showed that non‐specific binding of r‐proteins to the resin played a minor role, as 19 proteins from non‐transgenic controls were annotated as r‐proteins (median PSM value of 2). They were proteins from RPS3, RPS5, RPS7 and RPS20 families. The corresponding proteins were repeatedly detected in the ribosome samples purified from FLAG‐RPL18 plants, but with higher PSM scores, and they were therefore included in the riboproteome analysis (Table S5, see Supporting Information). Non‐transgenic controls had no hits for RPL proteins (Table S4). The most abundant contaminants that were repeatedly detected with high PSM scores were cytoplasmic proteins for elongation factor 1 for which α, β, δ and γ subunits were detected, E3 ubiquitin‐protein ligase HERC2‐like and clathrin interactor EPSIN 2 (Table S4). We also detected multiple hits for peroxisomal catalase isozyme 1 and plastid proteins trigalactosyldiaglycerol 2, ribulose bisphosphate carboxylase/oxygenase activase 2, carbonic anhydrase, ribulose bisphosphate carboxylase small chain 8B and phosphoglycerate kinase (Table S4). In addition, a few non‐annotated proteins, such as NbS00006811g0211 and NbS00003380g0115, were relatively abundant in the control samples. The PSM values for these non‐specifically bound proteins varied in the range 2–42, with the median value being 3. In general, proteins present in non‐transgenic control samples were regarded as non‐specific binders and were excluded from the analysis of putative ribosome‐associated proteins (see below).

### Total number of r‐protein paralogues incorporated into *N. benthamiana* ribosomes is the lowest in PVA‐infected samples

Next, we wanted to determine how versatile is the set of different r‐protein paralogues assembling in *N. benthamiana* ribosomes. We studied the protein composition of *N. benthamiana* ribosomes by analysis of the LC‐MS/MS identified peptides derived from 6555 r‐protein hits (Tables 1 and S5). Thus, more than one‐half of the obtained hits were r‐proteins. The detected r‐proteins presented 424 distinct r‐proteins (Tables  1 and S5) from 71 of the 81 r‐protein families. As expected, the bait was identified in all FLAG‐RPL18 samples. The total numbers of r‐protein hits in differently treated samples were as follows: healthy, 2272; *Agrobacterium*‐infected, 2360; PVA‐infected, 1923 (Table 1). The PSM values varied in the range 2–32, but values of 3 and 4 were most common. One to 12 peptides were identified for each r‐protein hit and the median value was 2. Sequence coverage varied from ~3% to 92%, the average being ~20%.

**Table 1:** Summary of riboproteome mass spectrometry (MS) data. Median values, averages and range are shown for sequence coverage (%), number of identified peptides and peptide matching scores (PSM). Data include r‐proteins (ribosomal proteins), P‐proteins and RACK (ribosome‐associated receptor for activating C kinase) having PSM values above 1.

	Mock	*Agrobacterium*‐infected	Potato virus A‐infected
Total number of r‐protein hits	2272	2360	1923
Unique r‐protein hits	417	421	384
Median coverage (%)	18.7	18.8	16.7
Average coverage (%)	20.4	20.8	18.4
Coverage range (%)	3.0–77.9	3.0–92.0	1.8–72.6
Median number of peptides per protein	2.0	2.0	2.0
Average number of peptides per protein	2.7	2.8	2.4
Number of peptides per protein: range	1–12	1–12	1‐10
Median PSM value	4.0	4.0	3.0
Average PSM value	5.3	5.3	3.9
Range for PSM values	2–27	2–32	2–20

Our riboproteome data contained 813 r‐proteins that were identified on the basis of unique peptides. These peptides derived from 97 distinct paralogues from 45 r‐protein families (Table S2, proteins marked in italics). Ribosome samples allowed the identification of 87 paralogues from healthy plants, 88 from *Agrobacterium*‐infected plants and 69 from PVA‐infected plants (Table S5). Approximately every tenth r‐protein identification (6555/813) was paralogue specific, and each paralogue was identified approximately eight times (813/97); however, in practice, some paralogues were repeatedly detected and some only occasionally. The number of identified unique peptides per paralogue varied from one to four, but one paralogue‐specific peptide was the most common. The average sequence coverage, PSM value and number of identified peptides per paralogue were ~27%, ~7 and ~4, respectively. From FLAG‐RPL18 samples, those being PVA infected had the lowest number of paralogues in their ribosome structure.

### Detection of 175 distinct r‐proteins of the 40S subunit representing 31 r‐protein families from *N. benthamiana* ribosomes

**Figure 6:**
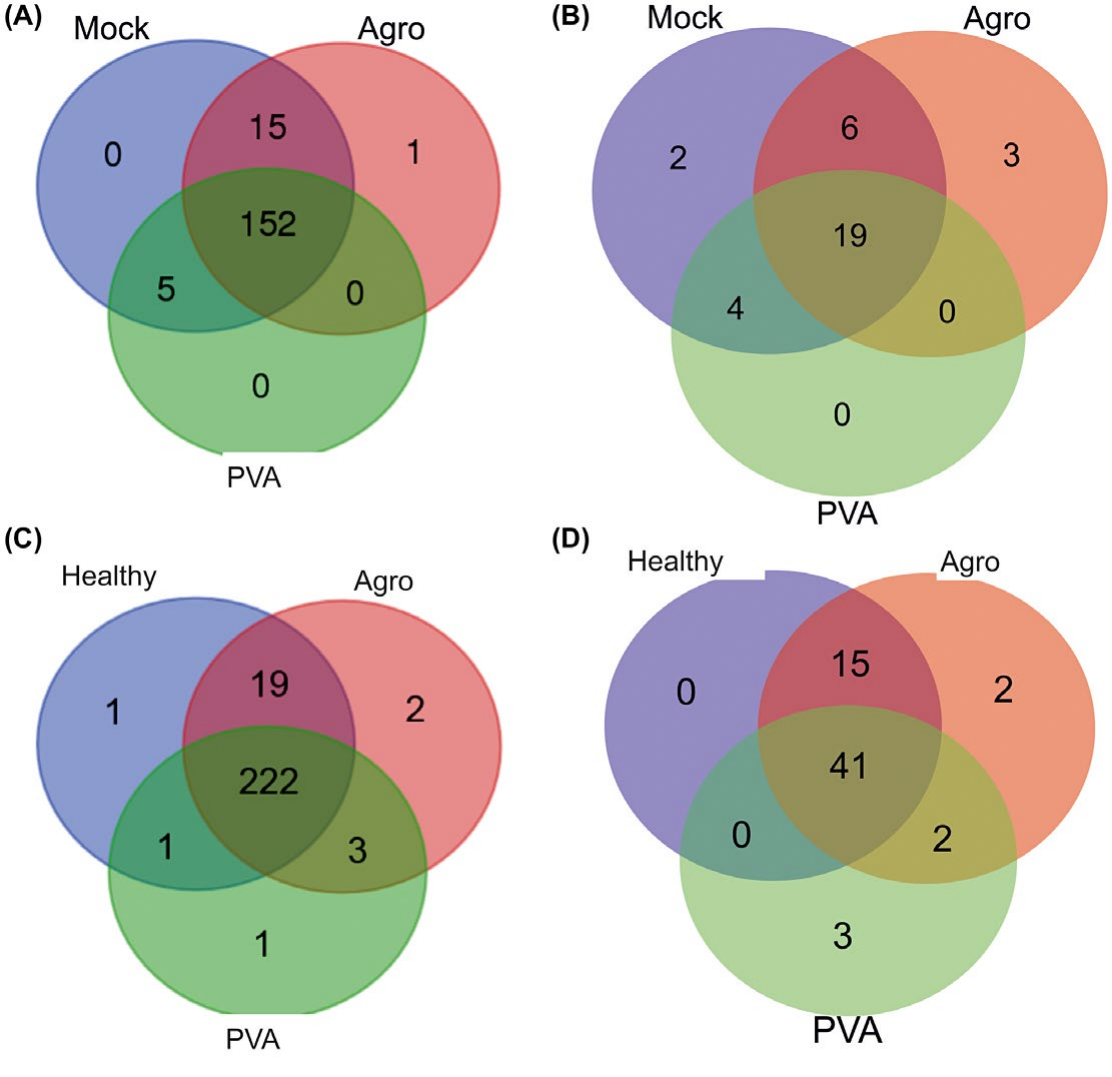
Venn diagrams for the identified ribosomal proteins (r‐proteins). (A) Unique 40S r‐proteins. (B) 40S r‐protein paralogues. (C) Unique 60S r‐proteins. (D) 60S r‐protein paralogues. Multiple hits for r‐proteins were removed. Agro, *Agrobacterium*‐infected; PVA, Potato virus A‐infected. [Colour figure can be viewed at wileyonlinelibrary.com]

We next scrutinized the presence of 40S subunit RPS proteins. The riboproteome data had 2986 hits that represented 175 distinct RPS proteins from 31 families (Fig. 6A; Table S2). Thus, each RPS protein was detected approximately 17 times (2986/175). We did not have hits for RPS25 or RPS29 within the filtered LC‐MS/MS data, but the non‐filtered original LC‐MS/MS data had a few hits for RPS25 (NbS00009478g0006, NbS00024925g0012 or NbS00017796g0009) and one for RPS29 (NbS00022995g0012) (Table S2, marked with asterisks). Ribosomes from healthy plants had 174 RPS protein identifications, whereas those from the *Agrobacterium*‐ and PVA‐infected plants had 175 and 159, respectively (Fig. 6A). The only difference in the RPS proteins present in ribosomes was the identification of RPS3a (NbS00010677g0005) in *Agrobacterium*‐infected samples. However, both PVA‐infected and healthy plants had hits for this protein in the non‐filtered MS data. Therefore, the conclusion is that r‐proteins of the same families formed the 40S subunits in both healthy and infected plants.

The combined riboproteome data of all FLAG‐RPL18 samples revealed 34 distinct RPS proteins that could be identified on the basis of unique peptides (Fig. 6B; Table S2, marked in italics). These paralogues represented 16 RPS families. Paralogue‐specific identification was most successful for healthy plants, which showed hits for 31 paralogues, whereas 28 and 23 paralogues were identified among the RPS proteins derived from ribosomes of *Agrobacterium*‐ and PVA‐infected plants, respectively. Single paralogues for RPS7 (NbS00048060g0002) and RPS8 (NbS00010815g0010) were specific for healthy plants. Three paralogues that encoded RPS3 (NbS00035854g0012), RPS8 (NbS00007872g0027) and RPS11 (NbS00030903g0006) were specific for *Agrobacterium*‐infected plants. No paralogue‐specific RPS protein identifications were made in PVA‐infected samples (Fig. 6B). In general, the treatment‐specific paralogues were detected on the basis of a limited number of hits (Table S5). Therefore, the identification of potential changes in RPS protein composition on infection requires further studies.

### Detection of 250 distinct r‐proteins of the 60S subunit representing 40 r‐protein families from *N. benthamiana* ribosomes

When the 60S subunit RPL proteins were examined more closely, a total of 3566 hits for RPL proteins that represented 250 distinct proteins (Table 1; Fig. 6C) from 40 families was found. Members of RPL29, RPL36, RPL36a, RPL37, RPL39 and RPL40 were not identified (Tables S2 and S5). When we looked for these proteins in the original non‐filtered LC‐MS/MS data, we found a few hits for RPL36, RPL36a, RPL37 and RPL40 in the FLAG‐RPL18 samples, but not in the non‐transgenic control purifications (Table S2, marked with asterisks). No hits for RPL29 or RPL39 were present in the non‐filtered MS data. As we did not have a reliable RPL41 sequence, we did not analyse this.

Ribosomes from healthy plants showed 244 identifications for unique RPL proteins, whereas those from *Agrobacterium*‐ and PVA‐infected plants showed 248 and 227, respectively (Table S5). The observed pathogen‐specific differences in RPL composition were the identification of RPL7a (NbS00034729g0013), RPL14 (NbS00006379g0022) and RPL37a (NbS00007843g0209) in *Agrobacterium*‐infected samples (Fig. 6C). No PVA‐specific identifications were made, but two RPL14 proteins (NbS00025105g0019 and NbS00038226g0005) and RPL21 (NbS00006309g0001) were specific for ribosomes of *Agrobacterium‐* and PVA‐infected plants. Ribosomes from healthy plants contained RPL30 (NbS00002468g0001) which was specific for them.

Altogether, 63 unique RPL proteins that represented 25 families had unique peptides enabling paralogue‐specific identifications (Table S2, marked in italics). Healthy, *Agrobacterium*‐ and PVA‐infected plants had 56, 60 and 46 paralogue‐specific identifications, respectively (Fig. 6D). Only infected plants had paralogues that were treatment specific. Two such paralogues were found from both *Agrobacterium*‐ and PVA‐infected plants. They encoded RPL6 (NbS00002188g0022) and RPL21 (NbS00006309g0001). Ribosomes from *Agrobacterium*‐infected plants contained two specific paralogues encoding RPL5 (NbS00021993g0016) and RPL14 (NbS00006379g0022). Ribosomes from PVA‐infected plants contained three specific paralogues that encoded RPL3 (NbS00038870g0030), RPL18 (NbS00020801g0014) and RPL27 (NbS00056842g0001). These findings enable us to propose that specific r‐protein paralogues may be incorporated into ribosomes as a result of *Agrobacterium *and PVA infections, but as their identification was based on a limited number of hits (Table S5), this is a matter to be studied further.

### Identification of novel phosphorylation events in r‐proteins and viral nuclear inclusion protein b (NIb)

*Arabidopsis* RPS2, RPS6, RPL13, RPL29, RPP0, RPP1, RPP2 and RPP3 are phosphorylated (Carroll, 2013). We analysed the putative phosphoproteins from our *N. benthamiana* riboproteome data. As we did not enrich phosphorylated proteins prior to our LC‐MS/MS analysis, we expected to observe only those phosphopeptides that were abundantly present. The whole dataset from FLAG‐RPL18 plants contained 1313 putative phosphoproteins: healthy, 507; *Agrobacterium*‐infected, 416; PVA‐infected, 390. *Arabidopsis* RPS6 is phosphorylated at a conserved C‐terminal site (Carroll *et al*., 2008; Chang *et al*., 2005; Turkina *et al*., 2011). We found two C‐terminal phosphopeptides for *N. benthamiana* RPS6 (Table 2). The conserved C‐termini of P‐proteins are also phosphorylated (Carroll, 2013), and there were hits for phosphorylated RPP1 and RPP2, together with a phosphopeptide that was common for both RPP2 and RPP3 in our data as well (Table 2). Phosphorylated P‐proteins were observed in the riboproteomes of *Agrobacterium*‐ and PVA‐infected plants, but not in healthy plants.

**Table 2:** Identified phosphopeptides. Phosphorylated amino acids marked in lower case.

Peptide sequence	R‐protein	Nb protein	Number of hits
SRLsAASKPSVAA	RPS6	NbS00006996g0012	1
SRLsAASKPSIAA	RPS6	NbS00012569g0012, NbS00000439g0011, Nbs00005003g0014, Nbs00002662g0109	2
KEEKEEsDDDMGFSLFD	RPP2, RPP3	NbS00021044g0016, NbS00005878g0110,	3
KEEPKEEsDDDMGFSLFD	RPP1	NbS00042976g0007, NbS00053503g0005	5
KVEEKEEsDDDMGFSLFD	RPP2	NbS00029619g0005, NbS00019361g0011	1
DyKSFRLFsLSIEDVNDK*	RPL18	NbS00020801g0014	1
SSsGTAPLIDVTQYGYFK	RPL27a	NbS00010663g0005	1
RFYAWVLEQsPyNALATTGLAPyIAESALK	PVA NIb		1

* N‐terminal extension before conserved methionine.

In addition to the previously known ribosomal phosphoproteins, we detected some novel ones. Phosphopeptides derived from RPL18 and RPL27a were detected. The phosphorylated peptide of RPL18 matched an N‐terminal extension of RPL18 preceding the conserved methionine (Table S3). This phosphoprotein was only detected in the riboproteomes of PVA‐infected plants. We did not detect the non‐phosphorylated form of the corresponding peptide. The phosphorylated peptide from RPL27a was located in the middle of the protein. The non‐phosphorylated peptide of RPL27a was also present in the riboproteome data. *Arabidopsis* RPL27a does not have the corresponding peptide sequence (Table S3). In addition, PVA NIb‐derived phosphopeptide was identified.

### Some non‐ribosomal proteins potentially associate with *N. benthamiana* ribosomes

Many non‐ribosomal proteins having an important regulatory role in translation, such as RACK1 and microRNA‐induced silencing complexes, associate with ribosomes. Therefore, the presence of non‐ribosomal proteins in the data was examined. We identified, in total, 540 hits for the non‐ribosomal proteins that were specific for samples purified from FLAG‐RPL18 plants (Table S6, see Supporting Information). These proteins presented 413 distinct proteins, 52 of which were identified on the basis of unique peptides. Most identified non‐ribosomal proteins co‐purified with the ribosomes only occasionally. Proteins that were repeatedly identified were a serine protease inhibitor 2 (seven identifications, NbC24872723g0001), F‐box family protein (four identifications, NbS00011177g0003), octanoyltransferase (11 identifications, NbS00020349g0003), pentatricopeptide repeat‐containing proteins (20 identifications, NbS00020849g0005) and malate dehydrogenase (five identifications, NbS00038595g0001).

Translation initiation and elongation factors are natural components of ribosomes. As elongation factors were non‐specifically purified (Table S4), their presence in the affinity‐purified ribosome could not be studied. We did not detect translation initiation factors in our riboproteome data either (Table S6). This was expected as most translation initiation factors are released on translation initiation and 80S formation, whereas affinity purification here was based on the enrichment of translationally active ribosomes via a protein of the 60S subunit.

### Unlike PVA proteins, proteins of *A. tumefaciens* do not associate with *N. benthamiana* ribosomes

We finally searched the riboproteome data against proteins of *A. tumefaciens *and PVA. For the six *A. tumefaciens* proteins identified in total, the PSM values were in the range 2–3 (Table S7, see Supporting Information). These bacterial proteins were also identified in the FLAG‐RPL18 samples from healthy plants (not infected with *Agrobacterium*) and in the non‐transgenic controls (no FLAG‐RPL18). In summary, we conclude that no *Agrobacterium*‐specific proteins were found to associate with the ribosomes.

PVA proteins HCPro and CI were detected in the riboproteomes derived from PVA‐infected transgenic plants at 4 dpi, but not in the samples collected at 3 dpi (Table 3). This might be a result of the logarithmic increase in the rate of virus multiplication between 3 and 4 dpi (Eskelin *et al*., 2010). In addition, NIb, NIaPro, VPg and CP were occasionally identified in the LC‐MS/MS data, but, in many cases, the PSM scores were below 2 (Table 3). No viral proteins were found from the virus‐infected, non‐transgenic control plants at 4 dpi, indicating that no non‐specific binding of the viral proteins to the affinity matrix occurred.

**Table 3:** Potato virus A (PVA) protein hits from affinity‐purified ribosomes at 4 days post‐infection (dpi). Peptide matching scores (PSM) are given.

	Purification 3, technical 1	Purification 3, technical 2	Purification 4, technical 1	Purification 4, technical 2
CI	11	8	2	3
HCPro	16	16	1	4
NIaPro	1	1	–	–
VPg	1	1	1	–
NIb	2	2	–	–
CP	1	1	1	1

## Discussion

This study reveals the riboproteome of *N. benthamiana* for the first time. Although the quality of the *N. benthamiana* genomic sequence data did not allow the recognition of all r‐proteins, the data obtained were sufficient to compare ribosomes of healthy and infected plants with the aim of pinpointing ribosomes carrying specialized r‐protein paralogues during pathogen infection. The comparison of the ribosome profiles and riboproteomes of healthy, *A. tumefaciens‐* and PVA‐infected *N. benthamiana* plants indeed revealed subtle changes. Although further studies are required to understand the functional relevance, some specific r‐protein paralogues of the 60S subunit were found only in the riboproteomes of *Agrobacterium‐* or PVA‐infected *N. benthamiana*. Interestingly, the number of r‐protein paralogues in ribosomes purified from PVA‐infected leaves was lower than that in those purified from either healthy or *Agrobacterium*‐infected leaves. As viruses are fully dependent on the host’s protein synthesis machinery, they have developed mechanisms to interfere with host translation. One mechanism proposed on the basis of this study is that PVA may fine tune the ribosome population to benefit its translation. Alternatively, the host may fine tune its ribosomes to limit viral protein synthesis. The association of the PVA proteins HCPro and CI with polysomes may indicate that potyviruses interfere with host protein synthesis via direct binding of its proteins to polysomes. None of the bacterial proteins associated with ribosomes specifically. This may mean that *Agrobacterium*, which has its own protein synthesis machinery, does not need to regulate host protein synthesis in a similar manner to viruses.

The studied pathogens did not induce major changes in the amount of translationally active polysomes or in the formation of 80S monosomes free of mRNA. Western blotting analysis revealed that PVA did not induce the cleavage of eIF4E/iso4E or PABP to repress host translation from capped mRNAs. These results are in agreement with previous studies. No differences could be detected in the total protein amount of PVA‐infected and healthy plants (Eskelin *et al*., 2011) or in the ribosome profiles of healthy and *Turnip mosaic virus* (TuMV, genus *Potyvirus*)‐infected leaves (Moeller *et al*., 2012). Along the same lines, the ribosome profiles of uninoculated *Medicago truncatula* roots and roots infected with *Sinorhizobium meililoti* belonging to the family *Rhizobiaceae* together with *Agrobacterium *were alike (Reynoso *et al*., 2013). Therefore, changes caused by these pathogens may occur in the composition of ribosomes, in the selective translation of specific mRNAs and/or in the specific localization of translating ribosomes, but not in the level of overall translational activity.

So far, the only riboproteomes of higher plants are those of cytosolic ribosomes of *Arabidopsis* leaves and cell suspensions (reviewed in Carroll, 2013). We identified approximately 660 putative r‐protein genes that represented 1–17 candidates from each r‐protein family. The 135‐Mb genome of *A. thaliana *contains 249 r‐protein genes that encode 2–7 paralogous r‐proteins from each r‐protein family (Barakat *et al*., 2001; Hummel *et al*., 2015). Internal variation among the r‐protein family members of *N. benthamiana* was higher than among the *Arabidopsis* counterparts. *N. benthamiana* sequence data contained r‐proteins that were incomplete or significantly different from the corresponding *A. thaliana *r‐protein sequences, whereas r‐proteins in general are rather conserved (Barakat *et al*., 2001). Therefore, it is likely that the accumulation of further sequence data will enable more accurate analysis and identification of the *N. benthamiana* r‐protein sequences and (ribo)proteomes.

We used FLAG‐tagged RPL18 to pull down ribosomes from *N. benthamiana *leaves. Translationally active polysomes complexed with various mRNAs were enriched in the samples and the 18S vs. 28S rRNA ratio remained similar in purified samples and total RNA samples. Subsequent LC‐MS/MS analysis resulted in comparable amounts of hits for r‐proteins of both subunits. Three other RPL18 paralogues were identified in addition to FLAG‐RPL18 in the riboproteomes. A previous comparison of *Arabidopsis* riboproteomes derived from ribosomes isolated by either affinity purification or ultracentrifugation showed no bias caused by the purification method (Hummel *et al*., 2015). Thus, we believe that the use of a single RPL18 paralogue in affinity purifications did not result in the enrichment of certain types of ribosomes. All of these facts indicated that translationally active high‐quality ribosomes had been purified.

LC‐MS/MS analysis provided us with approximately 6600 r‐protein hits that derived from 424 distinct r‐proteins. The sequence coverage for the identified peptides varied from ~3% to 92%, and was comparable with previously reported values for riboproteomes of *Arabidopsis*: 14%–70% (Chang *et al*., 2005), 7%–68% (Carroll *et al*., 2008) and 15%–89% (Hummel *et al*., 2015). We identified representatives from 71 of the 81 r‐protein families in the *N. benthamiana* riboproteome. However, the non‐filtered raw data contained hits for the two missing RPS protein families and five RPL protein families. We did not detect peptides for RPL29, RPL39 or RPL41. In *Arabidopsis*, RPL41 is currently the only r‐protein that has not been detected by proteomic approaches (Hummel *et al*., 2015). R‐proteins are rich in arginine and lysine residues, and trypsin digestion may generate peptides that are too small to be detected by LC‐MS/MS (Carroll *et al*., 2008). Therefore, we may have missed some of the *N. benthamiana* r‐proteins as a result of trypsination, and the use of alternative proteases could contribute to a more comprehensive dataset.

We recognized 97 paralogues that presented 43 r‐protein families of both subunits. Proteomic studies of cytoplasmic ribosomes of *Arabidopsis* have enabled the identification of 87 r‐protein paralogues with representatives from 45 RPS and RPL protein families (Carroll, 2013). The paralogue‐specific identifications of *N. benthamiana* and *A. thaliana* r‐proteins are listed in Table 4. Many of the identified paralogues were shared among *N. benthamiana* and *Arabidopsis,* but there were also differences. It is necessary to bear in mind that the paralogue‐specific identifications made in *N. benthamiana* were based on relatively few peptides and hits, preventing us from drawing solid conclusions with regard to the alterations in the ribosome’s r‐protein composition on infection. Therefore, it is necessary to study the functional relevance of these findings in future studies. It also remains to be studied whether more r‐proteins and their paralogues can be identified from other tissues, e.g. reproductive *N. benthamiana* tissues, which have more complex r‐protein transcript populations than the relatively quiescent leaf tissues (Whittle and Krochko, 2009).

**Table 4:** A comparison of the r‐protein paralogues identified from *Nicotiana benthamiana* and *Arabidopsis thaliana*.

*N. benthamiana* 40S	*A. thaliana* 40S*	*N. benthamiana* 60S	*A. thaliana* 60S*
RPS3a, RPS3, RPS4, RPS5, RPS6, RPS7, RPS8, RPS10, RPS11, RPS12, RPS14, RPS16, RPS17, RPS24, RPS27 and RACK1	RPSa, RPS2, RPS3a, RPS3, RPS6, RPS7, RPS10, RPS11, RPS12, RPS14, RPS15a, RPS15, RPS16, RPS19, RPS21, RPS23, RPS24, RPS25, S27a	RPP0, RPP1, RPP2, RPL3, RPL4, RPL5, RPL6, RPL7, RPL8, RPL9, RPL10, RPL11, RPL12, RPL13, RPL14, RPL15, RPL18, RPL19, RPL21, RPL22, RPL26, RPL27a, RPL27, RPL30, RPL34, RPL35, RPL38	RPP0, RPP1, RPP2, RPL4, RPL5, RPL6, RPL7, RPL7a, RPL8, RPL10, RPL10a, RPL13a, RPL17, RPL18a, RPL18, RPL19, RPL22, RPL23a, RPL26, RPL27, RPL28, RPL31, RPL32, RPL35, RPL36, RPL37a

* *Arabidopsis* heterogeneity in r‐proteins incorporated into ribosomes is as in Carroll (2013).

A large number of non‐ribosomal proteins have been identified in all published *Arabidopsis* riboproteomes (Carroll *et al*., 2008; Chang *et al*., 2005; Giavalisco *et al*., 2005; Hummel *et al*., 2012, 2015). Some may be true ribosome‐associated proteins and some may be co‐purifying contaminants. RACK1, eIF6A, subunits of the 20S proteasome and ferritin are the best candidates for true ribosome‐associated proteins (Carroll, 2013). For *N. benthamiana* ribosomes, the presence of four RACK1 paralogues was confirmed in all FLAG‐RPL18 samples, but the other identified candidates were novel with no obvious role in translation based on their annotation.

Virus infections can affect the phosphorylation status of r‐proteins and induce unusual phosphorylation events (Diaz *et al*., 2002). Our riboproteome data contained phosphopeptides for RPS6 in healthy and *Agrobacterium*‐infected ribosome samples only, but it has been shown recently that the levels of phosphorylated RPS6 are comparable in PVA‐ and TuMV‐infected and healthy *N. benthamiana* plants (Rajamäki *et al*., 2017). We found hits for phosphopeptides of RPP1 and RPP2 in the riboproteomes of *Agrobacterium*‐ and PVA‐infected plants, but not in healthy plants. Interestingly, we identified a phosphopeptide that belonged to the non‐conventional N‐terminal extension of RPL18 from ribosomes purified from PVA‐infected plants. PVA NIb, which is the viral RNA‐dependent RNA polymerase, has not been reported previously to be a phosphoprotein. Whether the detected phosphorylation event has any functional relevance remains to be studied.

Ribosomes from the PVA‐infected plants appeared to have a lower variety of r‐proteins incorporated (386) than the ribosomes purified from healthy (418) or *Agrobacterium*‐infected (423) plants. Potyviruses affect the transcription of r‐protein genes. Plum pox virus (PPV)‐ and TuMV‐infected plants show increased levels of several mRNAs encoding RPS and RPL proteins (Dardick, 2007; Yang *et al*., 2009). Interestingly, when transcript levels of the r‐protein paralogues were compared, differences in their accumulation were detected in PPV‐infected *N. benthaminana* plants, whereas no differences were observed in TuMV‐infected *A. thaliana* plants. Of the r‐protein transcripts, PVA infection increases the accumulation of the transcript encoding acidic r‐protein P0 in potato (*Solanum tuberosum* ssp. *andigena*) (Vuorinen *et al*., 2010), whereas infected *N. benthamiana* leaves show increased levels of rps6 mRNAs (Rajamäki *et al*., 2017). Potyvirus‐induced effects on r‐proteins at a protein level have been less well studied. Soybean mosaic virus infection represses the accumulation of both RPS12 mRNA and protein (Yang *et al*., 2011), and PVA and TuMV infections induce the accumulation of RPS6 (Rajamäki *et al*., 2017). Importantly, potyvirus infections are sensitive to the silencing of several *rps* and *rpl* genes (Rajamäki *et al*., 2017; Yang *et al*., 2009). Although silencing of P0 affects PVA infection substantially, this defect may relate mostly to the non‐ribosomal functions of P0 during infection (Hafren *et al*., 2015). In general, it appears that potyviruses differ in their effects on the ribosome composition or in the pool of r‐proteins displaying extra ribosomal functions, and that these effects may also vary in different host plants.

We found that potyviral proteins HCPro and CI, and potentially also NIaPro, VPg, NIb and CP, associated with the ribosomes, and that both HCPro and CI were bound to polysomes. No *Agrobacterium*‐derived proteins were detected in the riboproteomes. Interestingly, tagged Tobacco etch virus (TEV; genus *Potyvirus*) P1 has been shown to pull down 15 RPL proteins, RPS6 and RPS23 (Martinez and Daros, 2014). TEV P1 associates with 80S ribosomes and polysomes, and its interaction takes place via the 60S subunit (Martinez and Daros, 2014). PVA P1 was not present in our riboproteome data, suggesting that PVA and TEV may differ in their interactions with the host ribosomes. Affinity purification and MS identification of host proteins that interacted with TEV NIa showed that NIaPro part interacts with RPS3, RPL9 and RPL12, whereas VPg binds RPS12, RPS14 and RPL14 (Martinez *et al*., 2016). The currently known host proteins directly interacting with PVA CI or HCPro do not include any r‐proteins. However, r‐proteins have been identified by LC‐MS/MS from HCPro‐induced RNA granules, HCPro‐containing high‐molecular‐weight complexes and the viral replication complexes isolated from PVA‐infected *N. benthamiana* plants (Hafren *et al*., 2015; Ivanov *et al*., 2016; Lohmus *et al*., 2016). In addition, it has been shown that PVA VPg co‐localizes with RPS6 in the nucleus and nucleolus (Rajamäki *et al*., 2017), and HCPro co‐localizes with P0 in PVA‐induced RNA granules (Hafren *et al*., 2015). Our riboproteome analysis focused on translating ribosomes that lacked translation initiation factors, which are released from preinitiation complexes on 80S formation (Unbehaun *et al*., 2004). HCPro interacts with eIF4E/iso4E and CI (Guo *et al*., 2001), whereas CI interacts with VPg and eIF4E (Tavert‐Roudet *et al*., 2012). Thus, even though we cannot rule out the possibility that the interaction with eIF4E would bring HCPro and CI to the ribosomes, both need to stay in contact with the translationally active ribosomes via some other host protein(s), which may well be r‐proteins. All of these data indicate that several PVA proteins form complex interaction networks with components of the translation apparatus. Some of these interactions may be involved in the regulation of translation and some in extraribosomal functions. For example, TEV HCPro inhibits protein translation *in vitro* (Martinez and Daros, 2014), whereas P0, VPg and eIF4E/eIF(iso)4E promote PVA translation *in planta* (Eskelin *et al*., 2011; Hafren *et al*., 2013). Recently, we have demonstrated that HCPro and ARGONOUTE 1 interact with each other and are both associated with ribosomes, and propose that this may be required to release RNA silencing‐based translational repression of PVA RNA (Ivanov *et al*., 2016). Thus, several potential protein–protein interactions may bring the ribosomes and the potyviral proteins together. It will be interesting to identify the binding partners for PVA proteins on ribosomes and functionally characterize their role in PVA translation.

In summary, the findings of this study open up new and interesting avenues for research in ribosome heterogeneity during biotic and abiotic stress in *N. benthamiana*, the important model plant of plant pathology.

## Experimental Procedures

### Plant materials and growth conditions

Non‐transgenic and transgenic *N. benthamiana* plants expressing 35S‐FLAG‐RPL18B of *A. thaliana* (referred to here as FLAG‐RPL18) were grown at 22 ºC and 50% relative humidity under a 16‐h photoperiod and an 8‐h dark period in an environmentally controlled glasshouse. The two homozygous lines (2e and 6j) were a kind gift from Professor Peter Moffett (Université de Sherbrooke, QC, Canada).

### Infection of plants

Plants were infiltrated at the four‐leaf stage (~4–5 weeks old) with *A. tumefaciens* carrying *35S‐fluc‐nos* binary expression construct or *A. tumefaciens* harboring an infectious cDNA (icDNA) clone of PVA (Eskelin *et al*., 2010) (Fig. 1A). Here, we refer to this virus as wild‐type PVA (PVA^wt^). In some P170K preparations, PVA^wt^ that expressed HCPro fused to RFP was used (Hafren *et al*., 2015) to enable HCPro detection with RFP antibodies. For riboproteome studies, four independent plant batches were used. PVA^wt^ was used in riboproteome studies. Whole leaves corresponding to the same position in the plants were infiltrated. To achieve synchronous infection at all cells, high‐density *A. tumefaciens* suspensions [optical density at 600 nm (OD_600_) = 0.5] were used for infiltrations. With this density, the T‐DNA was transferred to all cells of the infiltrated area (Eskelin *et al*., 2010). A subset of plants was left untreated to represent healthy plants. We also infected non‐transgenic plants with PVA^wt ^to serve as control for non‐specific purification (Fig. 1A). Whole leaves were collected at 3 dpi (purifications 1 and 2) or 4 dpi (purifications 3 and 4). After harvest, the leaf samples were immediately snap frozen in liquid nitrogen and stored at –70 °C.

### Purification of ribosomes

Ribosomes were isolated as in the previously published protocol for *Arabidopsis* with some modifications (Ivanov *et al*., 2016; Zanetti *et al*., 2005). Frozen, pulverized leaf tissue (~4 mL) was homogenized with 1 vol of polysome extraction buffer [PEB: 200 mm Tris‐HCl (pH 9.0), 200 mm KCl, 36 mm MgCl_2_, 10 mm ethylene glycol‐bis(2‐aminoethylether)‐N,N,N′,N′‐tetraacetic acid (EGTA), 1 mg/mL heparin, 1 mm dithiothreitol (DTT), 50 µg/mL cycloheximide, 50 µg/mL chloramphenicol, 2% Triton X‐100, 2% Tween‐40, 2% polyoxyethylene (23) lauryl ether (Brij35), 2% nonylphenol ethoxylate (NP‐40), 2% polyoxyethylene 10 tridecyl ether (PTE), 1% deoxycholine] for 30 min at 4 °C. Homogenates were clarified by two subsequent centrifugations at 16 000 ***g*** for 10 min at 4 °C. Equal amounts of lysates were mixed with 50 µL of ANTI‐FLAG M2 affinity gel beads (Sigma‐Aldrich, USA) at 4 °C with gentle rotation for 1 h. After incubation, cleared resin was briefly rinsed with 1 mL of PEB, followed by incubation with PEB for 5 min at 4 °C. Next, the resin was washed three times for 5 min with 1 mL of washing buffer [WB: 40 mm Tris‐HCl (pH 8.8), 100 mm KCl, 10 mm MgCl_2_] at 4 °C. Ribosomes were eluted with WB that contained 200 ng/mL of 3×FLAG peptide (Sigma‐Aldrich) at 4 °C for 30 min. Eluted material was stored at –70 °C.

Crude ribosome preparations were obtained by pelleting the leaf extracts in sucrose cushions by ultracentrifugation at 170 000 ***g***, as described previously (Pitkänen *et al*., 2014; Zanetti *et al*., 2005). The obtained ribosome pellets (P170K) were rinsed and resuspended in ribosome resuspension buffer [40 mm Tris‐HCl (pH 8.8), 10 mm MgCl_2_, 1 mm DTT]. RNA and protein amounts in the ribosome samples were determined with an Eppendorf BioPhotometer (Eppendorf, Hamburg, Germany) based on absorption at 260 and 280 nm.

### RNA purification and RT‐PCR analysis

RNA was purified from pulverized leaves or immunopurified ribosomes using RNeasy columns (Qiagen, Hilden, Germany). RNA integrity was monitored with agarose gel electrophoresis and ethyl bromide staining. For RT‐PCR analysis, cDNA was synthesized using Superscript III (Invitrogen, Waltham, Massachusetts, USA) and random hexamers (MBI Fermentas, Vilnus, Lithuania) according to the manufacturer’s recommendations. The primers used were specific for bZIP60, P0 protein, varicose, casein kinase II and PABP (Table S1, see Supporting Information). DNA amplification was performed using Dynazyme II DNA polymerase (Thermo Fisher Scientific, Schwarte, Germany) according to the manufacturer’s recommendations. The 250–500‐bp PCR products were analysed in agarose gels that were stained with ethyl bromide.

### SDS‐PAGE and western blotting

Proteins were separated on in‐house made 15% (w/v) SDS‐PAGE gels or in Any kD Mini‐Protean TGX precast gels (BioRad, Hercules, CA, USA), and stained with silver or subjected to immunoblot analyses. For immunodetection, proteins were transferred to polyvinylidene difluoride (PVDF) membrane (Millipore, USA) and blocked with PBST (1 mm Na_2_HPO_4_, 0.14 mm KH_2_PO_4_, 13.7 mm NaCl, 0.27 mm KCl and 0.1% (v/v) Tween‐20) that contained 3% (w/v) non‐fat milk powder for 1 h. Blots were washed with PBST and incubated with PBST that contained 1% (w/v) non‐fat milk powder and the antibody of choice. Purified antisera and mouse monoclonal anti‐RFP (SignalChem, Richmond, Canada) were used to detect PVA CI, CP, VPg/NIa and RFP‐tagged HCPro. Anti‐FLAG‐HRP (Sigma‐Aldrich) and anti‐RPS6 (Santa Cruz Biotechnology, USA) antibodies were used to detect FLAG‐RPL18 and RPS6. Antibodies against eIF4E, eIFiso4E, PABP, eIF4G and eIFiso4G were kind gifts from Professor Karen Browning (University of Texas, UT, USA). Horseradish peroxidase (HRP)‐conjugated anti‐rabbit or anti‐mouse secondary antibodies (Promega, USA) were used as secondary antibodies. Antibody–antigen complexes were detected by the addition of 3,3′, 5,5′‐ tetramethylbenzidine (TMB)‐stabilized HRP substrate (Promega) or enhanced chemiluminesence (ECL) reagent (Millipore) to the membrane, followed by colour development or exposure to Kodak (Sigma Aldrich, Saint Louis, MO, USA) Biomax films.

### MS and data analysis

The protein concentrations of samples were adjusted to be comparable based on *A*_260_ and *A*_280_ measurements. Cysteine bonds of affinity‐purified proteins were reduced with 0.05 m Tris(2‐carboxyethol)phosphine hydrochloride (TCEP, #C4706 Sigma‐Aldrich) at 37 ºC for 20 min and alkylated with 0.15 m iodoacetamide (#57670 Fluka, Sigma‐Aldrich) at room temperature. Samples were digested by the addition of 0.75 µg trypsin (Sequencing Grade Modified Trypsin, Promega). Afterwards, digestion peptides were purified with C18 microspin columns (Harvard Apparatus, Holliston, MA, USA) and redissolved in 30 µL of buffer A (0.1% trifluoroacetic acid and 1% acetonitrile in MS‐grade water).

Liquid chromatography coupled to tandem mass spectrometry (LC‐MS/MS) analysis was carried out on an EASY‐nLC (Thermo Fisher Scientific) connected to a Velos Pro‐Orbitrap Elite ETD hybrid mass spectrometer (Thermo Fisher Scientific) with nanoelectrospray ion source (Thermo Fisher Scientific). The LC‐MS/MS samples were separated using a two‐column set‐up consisting of a 2‐cm C18‐A1 trap column (Thermo Fisher Scientific), followed by a 10‐cm C18‐A2 analytical column (Thermo Fisher Scientific). The linear separation gradient consisted of 5% buffer B for 5 min, 35% buffer B for 60 min, 80% buffer B for 5 min and 100% buffer B for 10 min at a flow rate of 0.3 µL/min [buffer A: 0.1% formic acid (FA) and 0.01% trifluoroacetic acid (TFA) in 1% acetonitrile; buffer B: 0.1% FA and 0.01% TFA in 98% acetonitrile]. One sample (4 µL) was injected per LC‐MS/MS run. Each sample was analysed in duplicate. The analyses were performed in a data‐dependent acquisition mode using collision‐induced dissociation (CID). A full MS scan was acquired with a resolution of 60 000 at normal mass range in the Orbitrap analyser. The method was set to fragment the 20 most intense precursor ions with CID (energy 35). Data were acquired using LTQ Tune software (Thermo Fisher Scientific).

The calibrated peak files from the Orbitrap Elite were searched against the *N. benthamiana* protein sequence database (Niben.genome.v0.4.4.proteins.annotated.fasta) downloaded from the Sol Genomics Network (www.solgenomics.net) using SEQUEST. The fasta file was modified to include the PVA‐encoded proteins P1, HCPro, 6K1, CI, 6K2, VPg, NIa, NIb, Rluc and CP. Bacterial protein sequences were obtained from the complete sequence of *A. tumefaciens* strain C58 circular chromosome (accession numbers AE007869, AE007943–AE008196, AE008688 and AE008975–AE009230) (Wood *et al*., 2001 *Science*, **294**: 2317–2323). Error tolerances on the precursor and fragment ions were ±15 ppm and ±0.6 Da, respectively. Database searches were performed to tryptic peptides allowing a maximum of two missed cleavages. Carbamidomethyl cysteine and methionine oxidation or phosphorylation (S/T/Y) were set as fixed and variable modifications, respectively. For peptide identification, a false discovery rate (FDR) < 0.05 was used. Proteins with PSM equal to or above two were included in the analysis. For the identification of potential phosphoproteins, the whole riboproteome dataset was included. Outputs of the individual runs (four treatments, four purifications, two technical repetitions) were merged and filtered in Excel. First, we removed proteins that were identified with a PSM value equal to one. Second, proteins that were identified in the non‐transgenic control samples were removed from the FLAG‐RPL18 samples to exclude non‐specific binders from the analysis. The number of distinct proteins was obtained by removing multiple hits. Proteins were divided into r‐proteins and potential ribosome‐associated proteins on the basis of the annotations. r‐proteins were further subdivided into RPS and RPL proteins. Paralogues were identified on the basis of unique peptides.

### AF4 analysis of ribosomes

AF4 experiments were carried out using an AF2000 MT instrument and software (Postnova Analytics, Landsberg, Germany), as described recently (Pitkänen *et al*., 2014). Regenerated cellulose (RC) membrane with a molecular weight cut‐off (MWCO) of 10 kDa (Postnova Analytics) was used in combination with a 350‐μm spacer. Sample elution was monitored at 254 nm using a UV detector (Shimadzu SPD‐20A; Shimadzu, Kyoto, Japan). Samples were focused for 6 min, followed by a 1‐min transition to the elution phase, where the cross‐flow ramped linearly from 1.0 to 0.05 mL/min in 40 or 50 min. The channel flow was 0.2 mL/min and the channel temperature was 4 ºC. Ribosome resuspension buffer was used as the mobile phase. P170K samples from healthy and pathogen‐infected leaves were analysed with AF4. Aggregated material was removed by short centrifugation prior to loading (Eppendorf centrifuge 5415D, 10 000 ***g***, 5 min, 4 ºC). The stability of 80S ribosomes was studied by incubating P170K samples in 1 m KCl for 10 min prior to AF4. Retention times for ribosomal subunits and intact ribosomes were obtained from Pitkänen *et al*. (2014). Fractions were concentrated with Amicon ultracentrifugal filters having MWCO of 10 kDa (Millipore). Concentrates were run in Any kD gels and stained with silver or subjected to Western blotting, as described above.

### Bioinformatics

Annotated *A. thaliana *and *N. sylvestris *protein sequences were obtained from the National Center for Biotechnology Information (NCBI) ftp server (datasets: GCF_000001735.3_TAIR10_protein.faa and GCF_000393655.1_Nsyl_protein.faa, respectively). *N. benthamiana* protein sequences were obtained from the Sol Genomics Network database ftp server (dataset: Niben.genome.v0.4.4.proteins.annotated.fasta).

*Arabidopsis thaliana* 40S or 60S r‐proteins (Table S2) were selected as the initial r‐protein dataset (Barakat *et al*., 2001; Hummel *et al*., 2015). They were compared pairwise with all *A. thaliana *protein sequences using the local alignment program ‘water’ from the EMBOSS‐package (Rice *et al*., 2000), employing 80% similarity score as a cut‐off value to ensure that all r‐proteins were selected. Next, protein sequences from both *N. sylvestris *and *N. benthamiana* were compared pairwise with each individual r‐protein from *A. thaliana* using the program ‘water’ and 80% similarity score cut‐off value.

Similar sequences were clustered per plant per r‐protein family for further investigation. The program ‘CD‐HIT’ (Fu *et al*., 2012) was used to normalize the dataset: the length variation per r‐protein family was used to reduce the number of initial sequences in the dataset, as well as to remove overly similar sequences. CD‐HIT was run in three consecutive steps per plant per family to cluster 100%, 90% and, finally, 80% identical sequences by retaining the longest sequence in the cluster as the representative in each step.

Representative sequences were aligned separately per family using the multiple sequence alignment program ‘ClustalO’ (Sievers *et al*., 2011). The resulting trees were visualized using the program ‘Dendroscope’ (Huson and Scornavacca, 2012). L41 protein sequences were omitted from the final alignment and tree because of their short length compared with similar sequences from the other data. The mass spectrometry proteomics data have been deposited to the ProteomeXchange Consortium via the PRIDE (Vizcaíno *et al.*, 2016) partner repository with the dataset identifier PXD011602.

## Supporting information


**Fig. S1** AF4 profiles for non‐treated and KCl‐treated ribosomes of healthy and PVA‐infected *N. benthamiana* plants were compared to assay potential PVA‐induced changes in the accumulation of 80S monosomes free of mRNA. Ribosomes of A) healthy and B) PVA‐infected plants. C) Ribosomes of plants collected after 1 h heat shock at 45 ºC. Samples were focused for 6 min prior to elution using linearly decaying cross‐flow from 1 mL/min to 0.05 mL/min (dashed line). Elution of molecules was followed by monitoring UV detector intensity (V) at 254 nm (solid lines). Note the shorter elution program used in C. Retention times for sample components were obtained from Pitkänen et al. (2014).

**Fig. S2** Electron microscopy imaging of A) P170K ribosome preparations, B) FLAG‐affinity purified ribosomes, and C) control ribosomes from wheat germ extract *in vitro* translation mixture. Sample (3 µL, 10 × dilution in buffer) was pipetted on Glow discharged formvar‐carbon coated grids. After 30 s the grids were stained with 20 µL of 2% aqueous uranyl acetate by slowly pipetting the solution to the grid and at the same time continuously absorbing it at the opposite side of the grid with filter paper. Next, the grids were washed by dipping twice in distilled water droplet, the excess water was removed with filter paper and the grids were let to dry. The grids were observed at the same day using Jeol JEM‐1400 (Jeol Ltd., Tokyo, Japan) transmission electron microscope (80 kV).

**Fig. S3** Western blot analysis of eIF4E/eIFiso4E and PABP in *Agrobacterium*‐ and PVA‐infected plants. Samples collected at early time points of infection (1 and 2 dpi) showed no changes in the accumulation or in the size of PABP or eIF4E/eIFiso4E indicating that these initiation factors were not targeted by proteases. Total protein (~5 μg) was loaded per lane. Samples collected immediately after infection served as controls. The eIF4E blotting was performed by mixing antibodies detecting both isoforms with expected masses of ~25 kDa (eIF4E) and ~34 kDa (eIFiso4E).

**Fig. S4** Phylogenetic trees for ribosomal proteins of the small A), large (B) and acidic ribosomal proteins. The trees were visualized using program “Dendroscope” [Huson and Scornavacca: Dendroscope 3: An interactive tool for rooted phylogenetic trees and networks, Systematic Biology (2012).] L41‐protein sequences were omitted from the final alignment and tree due to their short length compared to similar sequences from the other data. Sequences are labelled as Sn_x_Am_f_l, where "Sn_x" is the compared sequence transcript with NCBI‐sequence‐id "NP_id" for *A. thaliana* and "XP_" for *N. sylvestris*. For *N. benthamiana* "Nb_id" is used. The "Am" is the for closest matching *A. thaliana* sequence. "f" refers to the family and "l" to the sequence length.

**Fig. S5** Affinity purified ribosomes are intact and associated with mRNA. A) Comparison of total RNA isolated from *N. benthamiana* leaves and affinity purified ribosomes. B) RT‐PCR analysis for the presence of host mRNAs in affinity purified ribosomes. C: positive PCR control using total RNA for cDNA synthesis, RT‐ first strand synthesis reaction without reverse transcriptase. P: PVA‐; A: *Agrobacterium*‐; and M: mock ‐infected healthy plants.

**Table S1** Primers used in RT‐PCR reactions.

**Table S2** *N. benthamiana* r‐proteins, their size distribution (in amino acids, aa) and homologies to *A. thaliana* and *N. sylvestris* r‐proteins as well as internal variation.

**Table S3** *N. benthamiana and A. thaliana *r‐protein sequences, length in amino acids and corresponding sizes in Da.

**Table S4** Protein hits from non‐transgenic control plants.

**Table S5** R‐protein hits from mock‐, *Agrobacterium*‐, or PVA‐ infected transgenic plants.

**Table S6** Non‐ribosomal protein hits from transgenic plants.

**Table S7** *A. tumefaciens* protein hits.
