# Supplementary figures and images for: Ribosome profiles and riboproteomes of healthy and Potato virus A‐ and Agrobacterium‐infected Nicotiana benthamiana plants
Source: Mol Plant Pathol. 2018 Dec 6;20(3):392–409. doi: 10.1111/mpp.12764 (PMC6637900; doi:10.1111/mpp.12764)

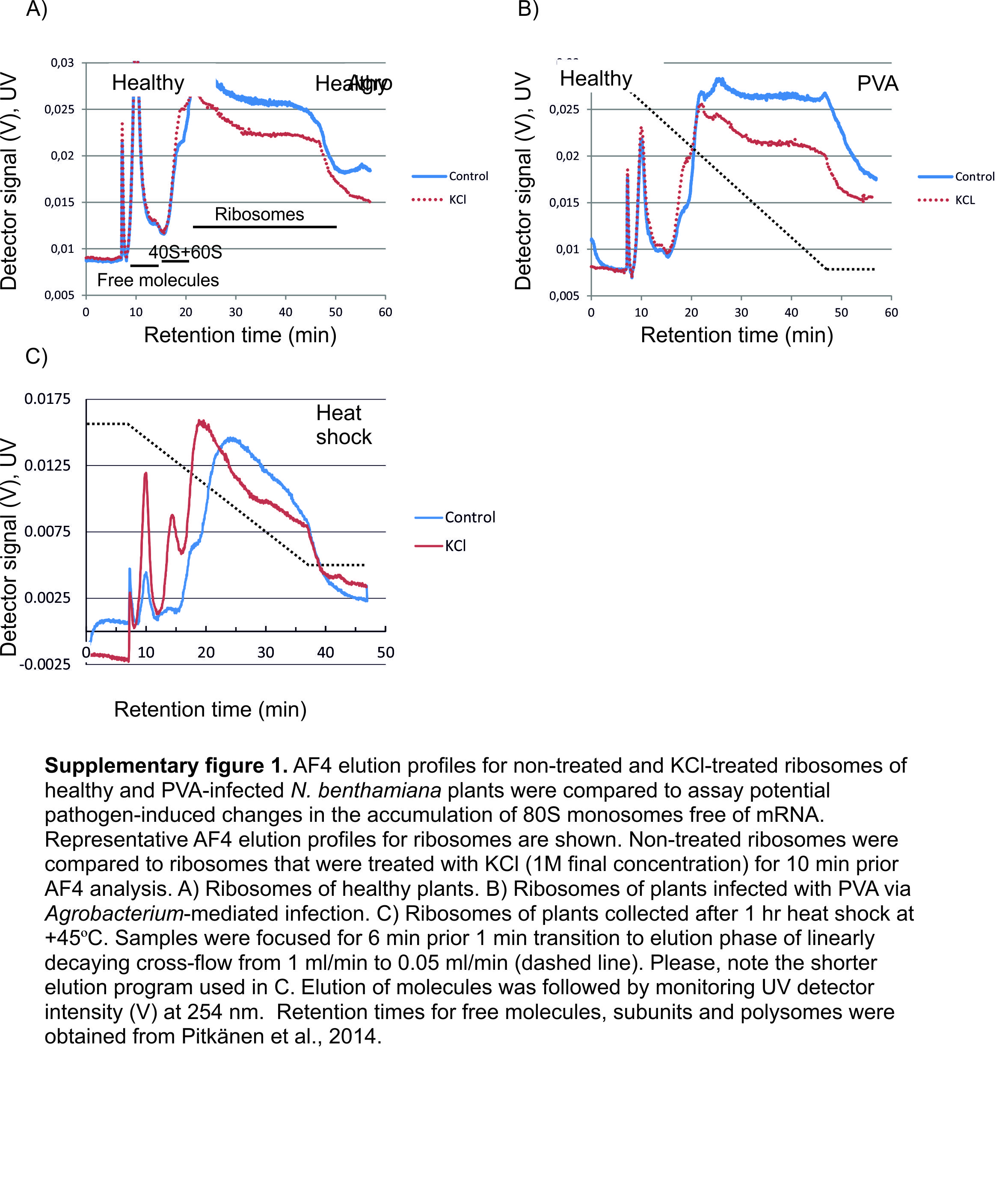

Supplement: Supplementary file 1 — Fig. S1 AF4 profiles for non‐treated and KCl‐treated ribosomes of healthy and PVA‐infected N. benthamiana plants were compared to assay potential PVA‐induced changes in the accumulation of 80S monosomes free of mRNA. Ribosomes of A) healthy and B) PVA‐infected plants. C) Ribosomes of plants collected after 1 h heat shock at 45 ºC. Samples were focused for 6 min prior to elution using linearly decaying cross‐flow from 1 mL/min to 0.05 mL/min (dashed line). Elution of molecules was followed by monitoring UV detector intensity (V) at 254 nm (solid lines). Note the shorter elution program used in C. Retention times for sample components were obtained from Pitkänen et al. (2014). [file MPP-20-392-s001.jpg]

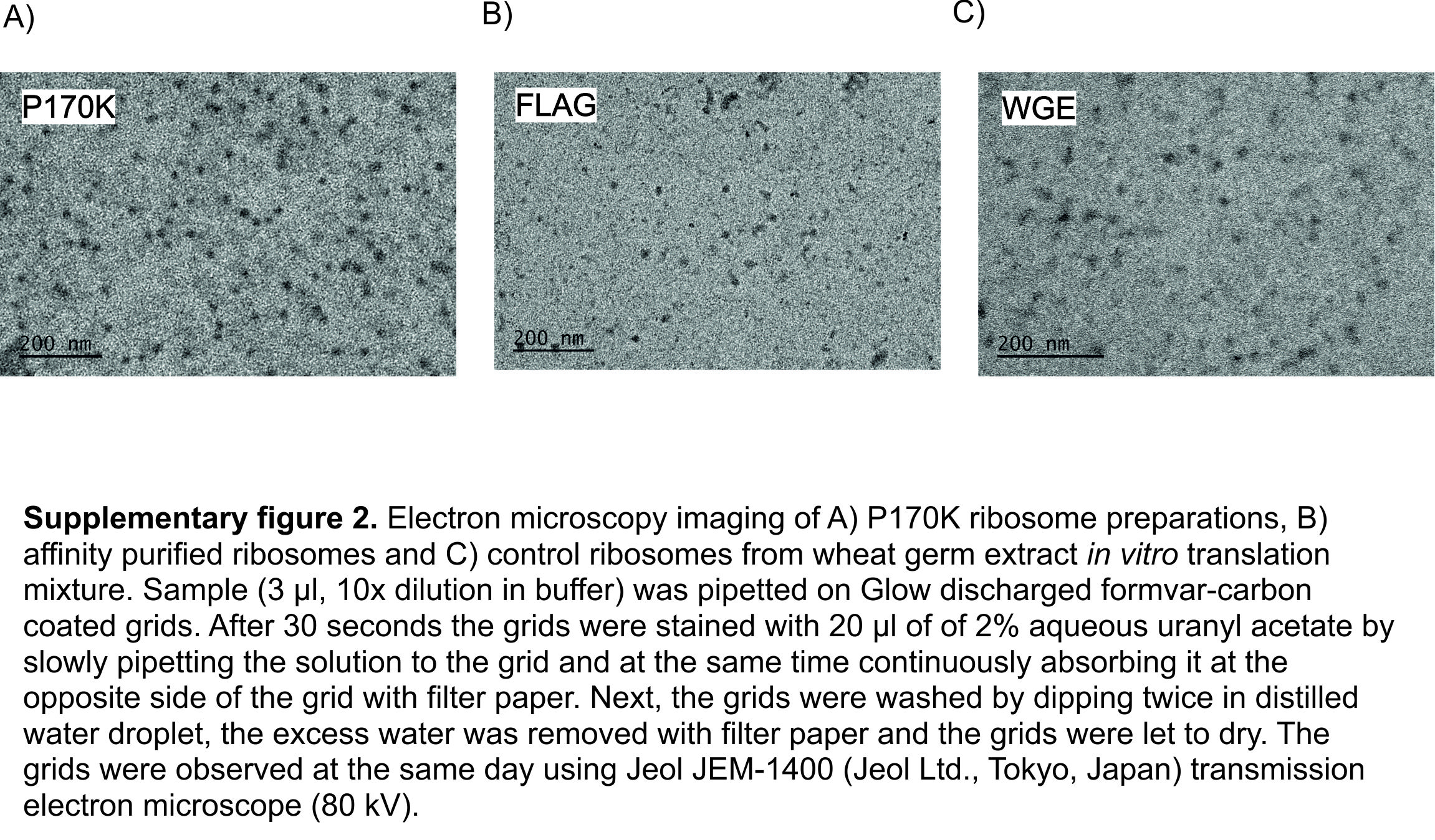

Supplement: Supplementary file 2 — Fig. S2 Electron microscopy imaging of A) P170K ribosome preparations, B) FLAG‐affinity purified ribosomes, and C) control ribosomes from wheat germ extract in vitro translation mixture. Sample (3 µL, 10 × dilution in buffer) was pipetted on Glow discharged formvar‐carbon coated grids. After 30 s the grids were stained with 20 µL of 2% aqueous uranyl acetate by slowly pipetting the solution to the grid and at the same time continuously absorbing it at the opposite side of the grid with filter paper. Next, the grids were washed by dipping twice in distilled water droplet, the excess water was removed with filter paper and the grids were let to dry. The grids were observed at the same day using Jeol JEM‐1400 (Jeol Ltd., Tokyo, Japan) transmission electron microscope (80 kV). [file MPP-20-392-s002.jpg]

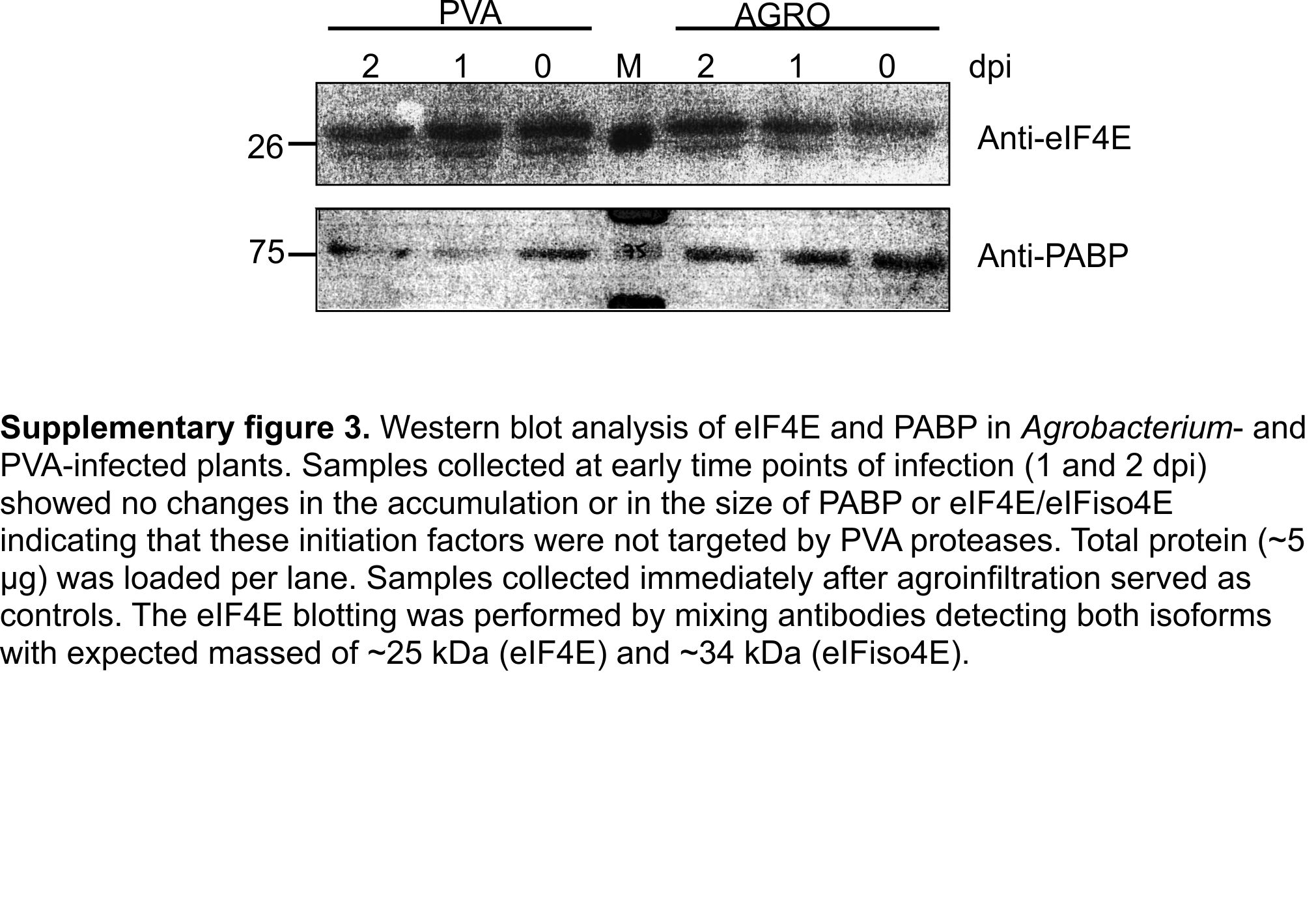

Supplement: Supplementary file 3 — Fig. S3 Western blot analysis of eIF4E/eIFiso4E and PABP in Agrobacterium‐ and PVA‐infected plants. Samples collected at early time points of infection (1 and 2 dpi) showed no changes in the accumulation or in the size of PABP or eIF4E/eIFiso4E indicating that these initiation factors were not targeted by proteases. Total protein (~5 μg) was loaded per lane. Samples collected immediately after infection served as controls. The eIF4E blotting was performed by mixing antibodies detecting both isoforms with expected masses of ~25 kDa (eIF4E) and ~34 kDa (eIFiso4E). [file MPP-20-392-s003.jpg]

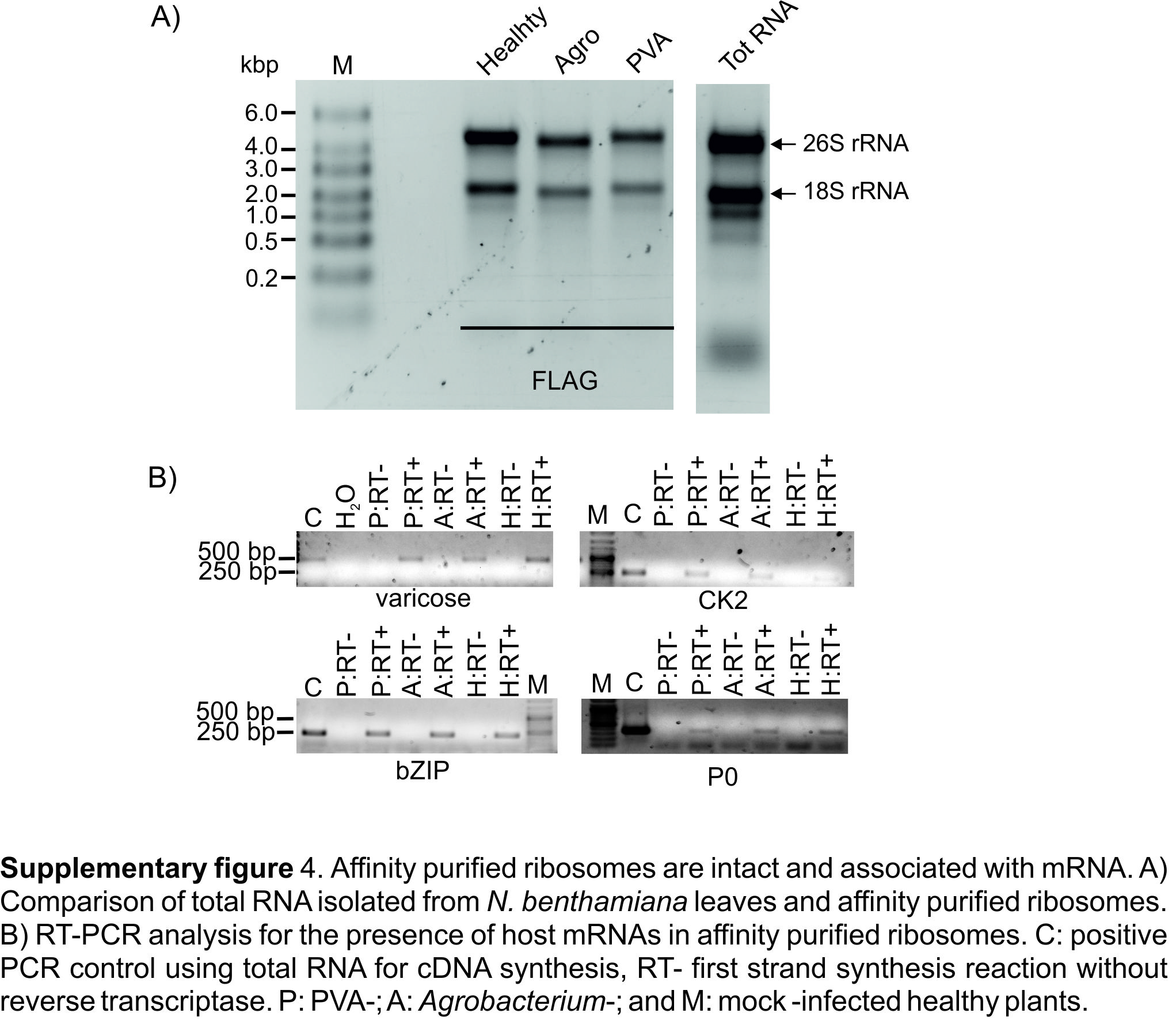

Supplement: Supplementary file 4 — Fig. S4 Phylogenetic trees for ribosomal proteins of the small A), large (B) and acidic ribosomal proteins. The trees were visualized using program “Dendroscope” [Huson and Scornavacca: Dendroscope 3: An interactive tool for rooted phylogenetic trees and networks, Systematic Biology (2012).] L41‐protein sequences were omitted from the final alignment and tree due to their short length compared to similar sequences from the other data. Sequences are labelled as Sn_x_Am_f_l, where "Sn_x" is the compared sequence transcript with NCBI‐sequence‐id "NP_id" for A. thaliana and "XP_" for N. sylvestris. For N. benthamiana "Nb_id" is used. The "Am" is the for closest matching A. thaliana sequence. "f" refers to the family and "l" to the sequence length. [file MPP-20-392-s004.jpg]
