# Supplementary material for: Ribosome profiles and riboproteomes of healthy and Potato virus A‐ and Agrobacterium‐infected Nicotiana benthamiana plants
Source: Mol Plant Pathol. 2018 Dec 6;20(3):392–409. doi: 10.1111/mpp.12764 (PMC6637900; doi:10.1111/mpp.12764)
Supplement: Supplementary file 8 — Table S1 Primers used in RT‐PCR reactions. [file MPP-20-392-s008.docx]

**Supplementary Table S1.** Primers used in RT-PCR reactions.

| **Primers** | **Primer sequences** |
| --- | --- |
| *P0*, fwd | 5´-ggatccgttggccattgctgttgagac-3´ |
| *P0*, rev | 5´-ggatccctaacacttgacaactgaaact-3´ |
| *bZIP60*, fwd | 5´-taaaaagcgcaagaggcaat-3´ |
| *bZIP60*, fwd | 5´-tcccaaataatggggattga-3´ |
| *varicose*, fwd | 5´-cagagagtcacagacatggctttctttgc-3´ |
| *varicose*, rev | 5´-ggcatagtgacagtaaactctgctatgtaatcca-3´ |
| *CK2*, fwd | 5´-cagaacctctgtgggggaccaac-3´ |
| *CK2*, rev | 5´-cattcctgcaaacatgcatccaag-3´ |
| *PABP*, fwd | 5´- aacccgggacaaaattccaaaatgtct-3' |
| *PABP*, rev | 5´- aacccgggatacaagttaagaccttggt-3' |
